# Dynamic-template-directed multiscale assembly for large-area coating of highly-aligned conjugated polymer thin films

**DOI:** 10.1038/ncomms16070

**Published:** 2017-07-13

**Authors:** Erfan Mohammadi, Chuankai Zhao, Yifei Meng, Ge Qu, Fengjiao Zhang, Xikang Zhao, Jianguo Mei, Jian-Min Zuo, Diwakar Shukla, Ying Diao

**Affiliations:** 1 Department of Chemical and Biomolecular Engineering, University of Illinois at Urbana−Champaign, 600 South Mathews Avenue, Urbana, Illinois 61801, USA; 2 Department of Materials Science and Engineering, University of Illinois at Urbana−Champaign, 104 South Goodwin Avenue, Urbana Illinois 61801, USA; 3 Department of Chemistry, Purdue University, 560 Oval Drive, West Lafayette, Indiana 47907, USA

## Abstract

Solution processable semiconducting polymers have been under intense investigations due to their diverse applications from printed electronics to biomedical devices. However, controlling the macromolecular assembly across length scales during solution coating remains a key challenge, largely due to the disparity in timescales of polymer assembly and high-throughput printing/coating. Herein we propose the concept of dynamic templating to expedite polymer nucleation and the ensuing assembly process, inspired by biomineralization templates capable of surface reconfiguration. Molecular dynamic simulations reveal that surface reconfigurability is key to promoting template–polymer interactions, thereby lowering polymer nucleation barrier. Employing ionic-liquid-based dynamic template during meniscus-guided coating results in highly aligned, highly crystalline donor–acceptor polymer thin films over large area (>1 cm^2^) and promoted charge transport along both the polymer backbone and the π–π stacking direction in field-effect transistors. We further demonstrate that the charge transport anisotropy can be reversed by tuning the degree of polymer backbone alignment.

## Abstract (web summary)

Controlling macromolecular assembly across length scales during solution coating of semiconducting polymers is still a challenge. Here the authors show a biomineralization inspired dynamic templating method that expedites polymer nucleation and assembly which allows for surface reconfiguration.

Template-assisted self-assembly has been an important approach for manufacturing a wide range of materials. Most commonly used are epitaxial templates with highly ordered, rigid surface structures designed for imposing lattice matching. Due to the stringent requirements on template structures, it remains challenging to design epitaxial templates for most molecular compounds. Living organisms, however, use an alternative approach that is in stark contrast to rigid templates, wherein assembly of highly ordered structures is directed by disordered but dynamic templates capable of surface reconfiguration—a strategy often observed in biomineralization^1^,^2^,^3^. Such surface reconfigurability enables cooperative interactions with the assembling media to attain a level of morphology control beyond that achieved using rigid (static) templates.

Conjugated polymers have demonstrated potential uses in a diverse range of applications from transistors, solar cells, thermoelectrics and light-emitting diodes to medical devices. Unlike traditional electronic manufacturing that requires high temperature and high vacuum, conjugated-polymer-based electronics are solution processable and can be printed at near ambient conditions to produce flexible, light-weight, biointegrated electronics at low cost and large scale^4^,^5^,^6^,^7^. The electronic properties of conjugated polymers are known to be highly sensitive to morphology parameters across all length scales^8^,^9^,^10^,^11^,^12^,^13^,^14^. Microscopically, it is well established that the extent of order in nanoscopic crystalline/aggregated domains, described by paracrystallinity, determines the rate of intermolecular charge hopping and limits the global charge transport^10^. Macroscopic domain alignment has also been shown to enhance charge transport by a few times to over an order of magnitude^13^,^15^,^16^,^17^. Although aligned polymer thin films have been achieved via mechanical rubbing^18^ or slow drying on grooved surfaces^15^,^19^, printed/coated polymer thin films frequently exhibit low degrees of alignment and poor molecular ordering^20^,^21^,^22^. We ascribe this phenomenon to the timescale mismatch between sluggish polymer crystallization and assembly (minutes to hours) and high-speed coating (seconds). Methods to address the kinetic mismatch issue include using a preaggregated polymer solution during bar-coating^12^ and employing liquid-crystalline polymers during meniscus-guided coating (MGC)^12^,^13^,^16^,^23^. Despite these advancements, material-agnostic methods for controlling morphology across multiple length scales at once during coating/printing is still needed not only to enable large-scale manufacturing of high-performance devices but also for elucidating charge transport mechanism in conjugated polymers.

In this work, we demonstrate the concept of dynamic templating compatible with large area solution coating for directing crystallization-triggered multiscale assembly of conjugated polymers. The dynamic templates are constructed from ionic liquid (IL) hosted in nanoporous media. Two donor–acceptor (D-A) polymers are studied, DPP2T-TT with diketopyrrolopyrrole acceptor and thienothiophene donor and PII-2T with isoindigo acceptor co-polymerized with bithiophene donor. Both polymers belong to families of high-performance D-A polymers under intense investigations recently^24^,^25^. For both systems, our method dramatically enhances thin film morphology from molecular to macro scale to yield highly aligned, highly ordered polymer thin films not attainable by simply varying the coating conditions and substrate surface chemistry. Molecular dynamic (MD) simulations suggest that the dynamic nature of the template is critical to the observed enhancement in morphology. We hypothesize that the dynamic template can adjust its surface chemistry and conformations to maximize favourable interactions, thereby lowering the free energy barrier to polymer crystallization. We note that, coincidentally, IL has been previously employed as a supporting media for mechanical compression of conjugated polymers to attain alignment^17^. This previous work is conceptually different from our approach, as the IL served solely as a passive free surface with low volatility to support the mechanical compression process.

## Results

### Dynamic template design

**Figure 1:**
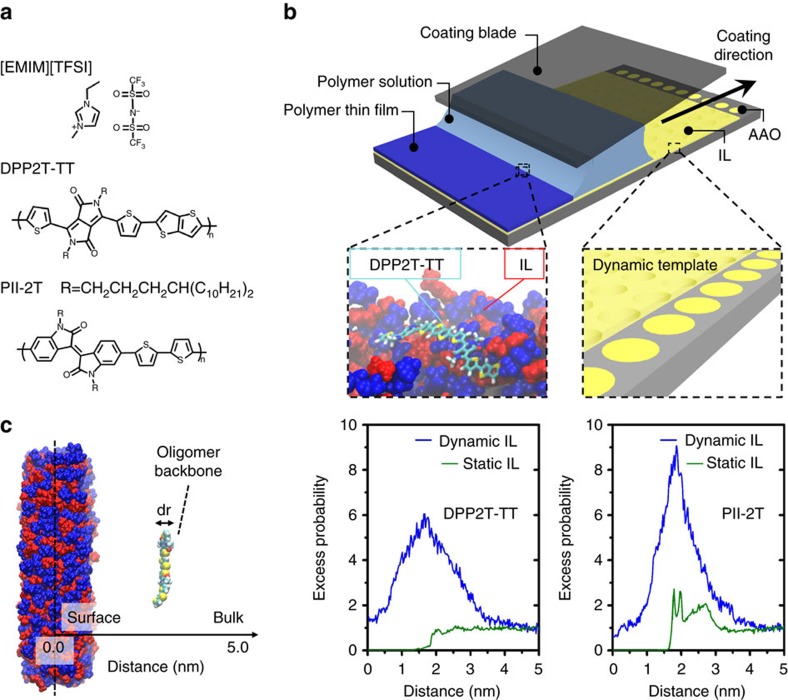
Dynamic template design for meniscus-guided coating and MD simulations of surface dynamic effect. (**a**) Molecular structures of ionic liquid [EMIM][TFSI] and conjugated polymers DPP2T-TT and PII-2T, where R is the alkyl chain for both polymers. (**b**) Schematic (not to scale) of MGC on the IL/AAO dynamic template. The black arrow indicates the coating direction. In the inset, part of the IL wetting layer is artificially removed to reveal the nanocomposite structure underneath. (**c**) Simulation box of the MD simulations and the calculated excess probability distributions with respect to the IL surface for dimeric DPP2T-TT and PII-2T, comparing the cases of dynamic versus static IL. Solvent molecules (chloroform) are omitted for clarity.

The molecular structures of the D-A polymers, DPP2T-TT and PII-2T, and the room temperature IL, 1-ethyl-3-methylimidazolium bis(trifluoromethylsulfonyl)imide ([EMIM][TFSI]), used in this study are shown in Fig. 1a. The dynamic template is constructed by infiltrating the IL in a nanoporous matrix, enabling us to implement solution coating on a liquid surface in the same fashion as on a solid substrate (Fig. 1b). Specifically, we used anodized aluminum oxide (AAO) with 200 nm through-pores as the porous host. We note that without the nanoporous matrix, MGC cannot proceed on a liquid surface, due to large surface deformation driven by the capillary force between the liquid and the coating blade. We verified experimentally that [EMIM][TFSI] is practically immiscible with the polymer ink solution in chloroform in the short time frame of solution coating, which is further supported by MD simulations (shown later). During coating, capillary force imparted by AAO nanopores retains the IL in place, while an IL-wetting layer forms on top of the substrate that separates the ink solution and the AAO host^26^. Moreover, we derived the stability criteria and verified that the IL-wetted hybrid template is energetically stable during solution coating owing to the high aspect ratio of AAO and the higher surface tension of IL relative to the ink solution (see Supplementary Note and Supplementary Figs 1–3). Since IL defines the top surface of the dynamic template, the IL–polymer interaction and IL surface dynamics are particularly important for directing polymer crystallization. We chose imidazolium-based IL considering its strong ion–π and π–π interactions with D-A polymers and its ultrafast surface dynamics reported before^27^,^28^. Ion–π and, in particular cation–π, interaction is among the strongest noncovalent interactions and is prevalent in biological systems^29^. In our case, large polarizability of D-A polymers may lead to particularly strong ion–π interactions with the IL. The interaction can be further strengthened by the electrostatic interactions between the cations with the strong electron-withdrawing groups in the acceptor unit (DPP) of the polymer backbone, likewise for anions with the donor unit (TT). We characterized IL–polymer interactions in the solvent environment using ^1^H NMR, which confirmed that the IL molecules favourably interact with the polymer backbone as opposed to the alkyl chains (Supplementary Fig. 4). MD simulation results further validated strong IL–polymer backbone interactions, discussed below. Regarding the surface dynamics of [EMIM][TFSI], large-scale rearrangements and collective motion of the cation and the anion were found to occur on the hundreds picosecond^27^ to one nanosecond timescale^28^, significantly shorter than the polymer crystallization timescale and the overall coating timescale set by solvent evaporation (seconds to minutes).

To validate our design concept and to investigate the effect of surface dynamics on IL–polymer interactions, we performed MD simulations with full atomic details and explicit solvents to realistically capture the molecular interactions between IL and polymers in the solution environment. Modelling the polymer crystallization process from solution is a daunting task given the huge size of the polymer, the long timescale and the stochastic nature of nucleation. Alternatively, we focus on determining the spatial distribution of conjugated molecules with respect to the IL surface in solution, as a measure of the extent of interactions between IL and conjugated polymers in the solvent medium. We normalize the distribution probability by the bulk value (defined as excess probability) to directly compare the spatial distribution of the conjugated molecule near the IL surface with that of the bulk. To reduce the computation time to a reasonable time frame, the simulations were performed using oligomers (monomers and dimers), and the alkyl chains were removed to capture the major interactions between IL and the conjugated core of the polymer. The simulation details are included in Supplementary Method. As Fig. 1c shows, the distribution probability of the dimers at the IL/chloroform interface is significantly higher than that in the bulk solution for both systems, with peak excess probability of 6 and 9 for DPP2T-TT and PII-2T, respectively. This result implies that the dimeric-conjugated molecules have strong favourable interactions with the IL surface, forming a concentrated layer near the interface. Distribution probability curves for the monomers also exhibit similar trends as shown in Supplementary Fig. 5, albeit with lower peak excess probabilities. To further investigate the influence of dynamic properties of IL on its interactions with the conjugated molecules, MD simulations were performed on the dimeric DPP2T-TT and PII-2T with and without position restraints on IL molecules. When the IL molecules were ‘frozen’, preferential association of the oligomer with the IL surface almost vanished with peak excess probabilities reduced to approximately 1 and 2 for DPP2T-TT and PII-2T, respectively (Fig. 1c). This result reveals the critical role of IL surface dynamics in promoting its interactions with the conjugated molecules, possibly by exposing favourable interaction sites and adapting surface structures in response to the association of conjugated molecules. Indeed, analysis of the IL radial distribution functions confirms that, in the presence of oligomers, IL significantly alters its surface structure to preferentially expose cations to the electronegative atoms in the conjugated backbone. While in the absence of oligomers, cations and anions are well mixed near the IL–solvent interface (Supplementary Fig. 6).

We acknowledge that approximating polymers with dimer backbones without alkyl chains is a limitation of our simulation study given the large system size. Although the side chains do influence the polymer–IL interactions by introducing entropic penalty, we expect that the strong multivalent interactions between long polymer chains and the IL will overcome the conformational entropy loss of the side chain^30^,^31^, when full size polymer chains are considered (18 monomer long). Our ^1^H NMR results (Supplementary Fig. 4) further verified that strong IL–polymer backbone interactions drive the association process, despite the presence of long alkyl chains.

### Dynamic-template-directed thin film morphology

We next investigate the effect of dynamic templates on multiscale morphology of solution-coated conjugated polymer thin films. We focus on the DPP2T-TT system for in-depth morphology characterizations and further validate the concept with a second polymer PII-2T towards the end of the paper. We prepared DPP2T-TT thin films via a MGC method^32^ (Fig. 1b) on both IL dynamic templates and static reference substrates. We employed octadecyltrichlorosilane (OTS) self-assembled monolayer functionalized SiO_2_ as the reference substrate, which is among the most commonly used dielectrics for fabricating organic field-effect transistors (OFETs) with low interfacial trap densities^33^. For direct comparison with the reference samples, films coated on dynamic templates were transferred to OTS/SiO_2_ substrates for morphology characterizations (see Supplementary Fig. 7 for detailed transfer process). We first qualitatively characterized the crystallinity and alignment of polymer thin films via cross-polarized optical microscopy (C-POM) and atomic force microscopy (AFM). We further quantified the degree of global and local alignment combining polarized ultraviolet–visible absorption spectroscopy, grazing incidence X-ray diffraction (GIXD) and large-area transmission electron microscopy (TEM) mapping. All experimental details are summarized in the Methods section.

**Figure 2:**
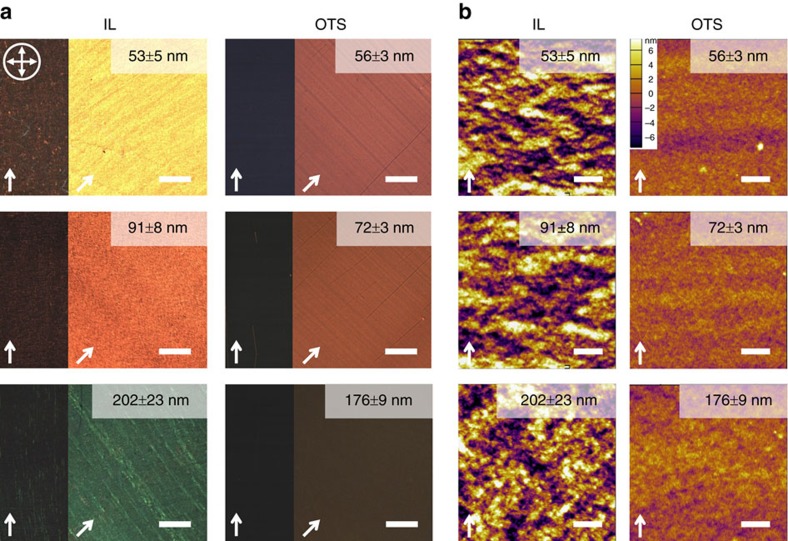
C-POM and AFM micrographs of IL-templated DPP2T-TT films versus reference films of various thicknesses. (**a**) Cross-polarized optical microscopic images. The orientation of the cross-polarizers is shown as crossed arrows, and the white arrows indicate the coating direction. All scale bars are 100 μm. (**b**) Tapping-mode AFM height images (scale bars are 1 μm). With increasing thickness, the films gradually lose in-plane orientation ordering and become isotropic. Film thickness is denoted on the upper right corner in all images.

By means of C-POM and AFM characterizations, we observed that IL dynamic template drastically increased crystalline domain size and possibly enhanced polymer global alignment across a wide range of film thicknesses studied. Optical birefringence provides a qualitative measure of the extent of global chain alignment. We observed total light extinction when the coating direction was aligned with either axis of the cross-polarizers (Fig. 2a), indicating that the conjugated backbone is oriented either parallel or perpendicular to the coating direction. Notably, higher birefringence of IL-templated films may arise from higher degrees of alignment and/or crystallinity compared to the reference films. AFM images show a dramatic change in mesoscale morphology induced by dynamic templates (Fig. 2b). The DPP2T-TT films coated on IL surface exhibit (semi)crystalline domains hundreds of nanometres wide and microns long, which are more than an order of magnitude larger than domains in the reference films. Such stark contrast in morphology persisted across the entire thickness of the film, suggested by AFM scans on both top and bottom surfaces of the polymer thin films (Supplementary Fig. 8). We also observed a pronounced domain orientational ordering in IL-templated films thinner than 100 nm. The orientation ordering was substantially enhanced when the film thickness was further decreased to <25 nm (Supplementary Fig. 9), corroborated with GIXD results discussed later. Interestingly, IL dynamic template enabled coating of highly aligned, highly crystalline ultrathin films of 5–10 nm not attainable on OTS substrates. Due to inadequate wetting, continuous films thinner than 35 nm could not form on OTS under the same conditions. We attribute this phenomenon to enhanced wetting on IL (Supplementary Fig. 10).

**Figure 3:**
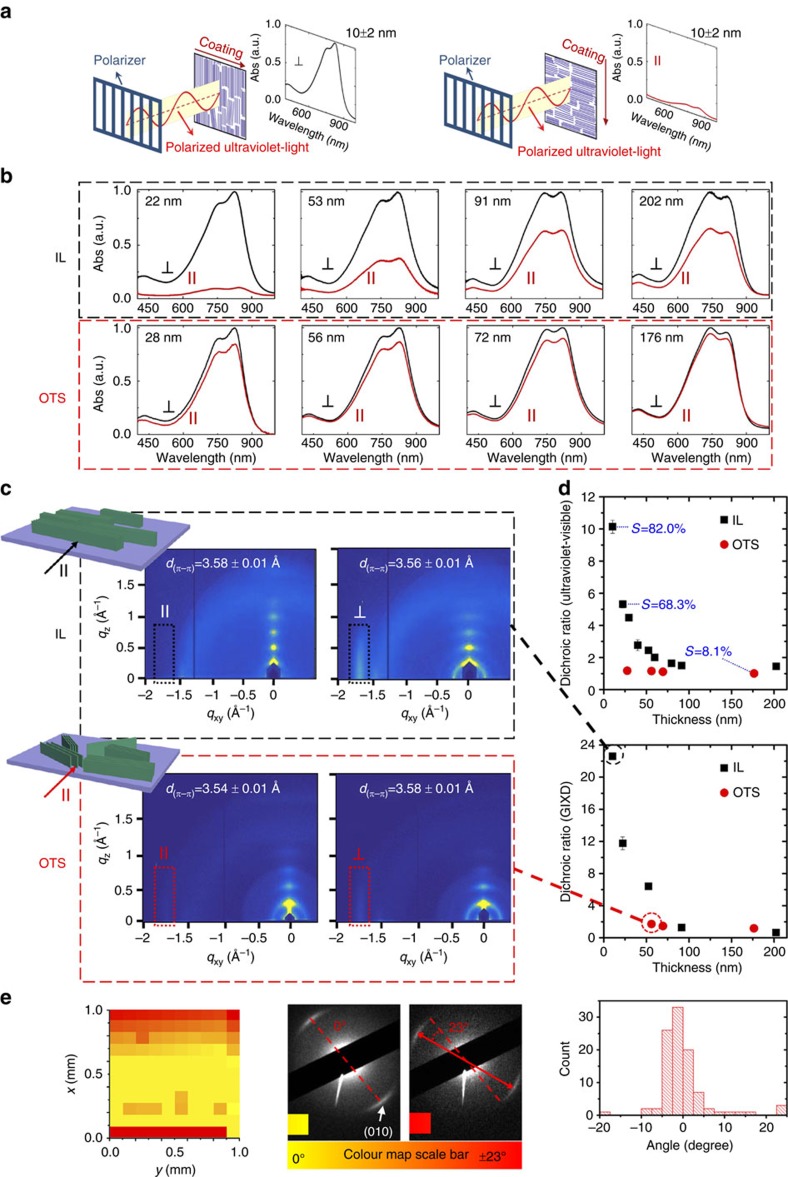
Quantifying global and local alignment in DPP2T-TT thin films. (**a**,**b**) Schematic and normalized absorption spectra of polarized ultraviolet–visible spectroscopy comparing DPP2T-TT films deposited on IL versus OTS. The sign ‖ (⊥) denotes the film orientation when the coating direction is parallel (perpendicular) to the axis of the polarizer. Average film thickness is denoted on the upper left corner of all spectra. (**c**) Comparison of GIXD images of films with the highest degree of alignment achievable on IL versus OTS substrates, with the incident beam oriented parallel and perpendicular to the coating direction. The dashed boxes highlight in-plane π–π stacking peaks. Schematics of thin film molecular packing are shown with the incident beam parallel to coating direction. More sharper lamella peaks were observed in IL-templated films, compared to fewer, broader lamella peaks observed in samples coated on OTS. (**d**) Ultraviolet–visible and GIXD dichroic ratios for films of various thicknesses. The orientation order parameter *S* was calculated from ultraviolet–visible dichroic ratio. (**e**) Orientation colour map from TEM diffraction, examples of diffraction micrographs (the most frequent, labelled as 0°, and the rarest, labelled as 23°, orientations are shown) and histogram of orientation distribution corresponding to the orientation colour map, respectively. The diffraction arcs represent DPP2T-TT π-π stacking peak (010) with d-spacing of 3.6±0.4 Å.

We next quantified the degree of global and local alignment in coated thin films using a combination of techniques (Fig. 3). We first applied polarized ultraviolet–visible spectroscopy to quantify global alignment of crystalline and amorphous regions combined using the dichroic ratio and the orientation order parameter (Fig. 3a,b). We next employed GIXD to characterize the global alignment of π-stacked crystalline domains specifically (Fig. 3c). We further mapped the local orientation distribution at a spatial resolution of 100 μm over an area of ∼1 mm^2^ using TEM electron diffraction (Fig. 3e).

In polarized ultraviolet–visible spectroscopy, maximum absorption is expected when the transition dipole moments align with the polarizer axis. We observed maximum absorbance with the coating direction positioned perpendicular to the polarizer orientation for all samples (Fig. 3b). This implies that the polymer backbone is preferentially oriented perpendicular to the coating direction, assuming that the transition dipole moment components of DPP2T-TT are oriented parallel to the polymer backbone^13^. The degree of alignment of the polymer backbone can be quantified using the 0-0 vibrational peak dichroic ratio, *R*=*I*_⊥_/*I*_‖_, where *I*_⊥_ and *I*_‖_ denote peak absorbance with the coating direction perpendicular and parallel to the polarizer, respectively. The dichroic ratio for the IL-templated films rose above that of the reference films across the whole range of film thicknesses tested, showing a striking enhancement in chain alignment (Fig. 3d). The highest dichroic ratio from IL-templated films reached *R*=10.1±0.4, corresponding to the thinnest film of 10±2 nm. This value is close to an order of magnitude greater than the dichroic ratios of the reference samples and is among the highest reported to date^13^,^16^,^19^. We further calculated the two-dimensional (2D) orientation order parameter, *S*=(*I*_⊥_−*I*_‖_)/(*I*_⊥_+*I*_‖_)^34^, with *S*=1 when the polymer chains are 100% aligned, and *S*=0 for isotropic morphology in-plane. In this work, the highest value of *S* reached 0.82 from the thinnest IL-templated film, validating the high degree of backbone alignment inferred from *R* (Fig. 3d). The degree of alignment decreased monotonically with increasing film thickness from 10±2 to 202±23 nm, consistent with diminishing birefringence from C-POM and lower orientation order from AFM (Fig. 2). This trend was coupled with the decrease in the 0-0/0-1 peak ratio and the blue shift of the 0-0 peak position from 845 to 810 nm (Supplementary Fig. 11). Taken together, we infer that the highly aligned thinner films exhibit stronger intrachain through-bond interactions or J-aggregation indicating more planar backbones, whereas the less aligned thicker films show reduced intrachain interactions and stronger interchain π–π interactions, characteristic of enhanced H-aggregation^35^. This phenomenon may be caused by the change in polymer conformation as a function of solution concentration.

The GIXD measurements corroborated the observations from ultraviolet–visible spectroscopy and revealed an even higher degree of alignment in the crystalline domains of the thin films. Figure 3c shows the GIXD patterns comparing films of the highest degrees of alignment achievable when coated on IL versus OTS. GIXD patterns of all samples and analysis are summarized in Supplementary Figs 12 and 13. In most cases, we observed a significantly more intense edge-on π–π stacking peak when the film was oriented with the coating direction perpendicular to the incident beam, confirming that the polymer backbone preferentially oriented perpendicular to the coating direction. To quantify the in-plane alignment, we define dichroic ratio as *R*=*A*_⊥_/*A*_‖_, where *A*_⊥_ and *A*_‖_ are the normalized edge-on π–π stacking peak area with the incidence beam perpendicular and parallel to the coating direction, respectively. The peak area was extracted following a multi-peak fitting procedure, accounting for background scattering and interference from the amorphous ring, and normalized by the irradiated volume as detailed in the Methods section. The calculated dichroic ratio reached as high as 22.6±0.1 for the thinnest IL-templated film, compared to 1.7±0.1 obtained from the highest aligned reference film on OTS (Fig. 3d). The GIXD dichroic ratio is significantly higher than that from the ultraviolet–visible, most likely since GIXD ‘sees’ the crystalline regions of the film, whereas ultraviolet–visible measures both amorphous and crystalline regions. Besides in-plane alignment, the out-of-plane ordering was also enhanced in IL-templated thin films. The highest-order lamella peak went up to (500) for IL-templated films, whereas fewer higher-order peaks were observed from the reference films (Fig. 3c). Additionally, we studied thickness-dependent anisotropy shown in Fig. 3d. Consistent with ultraviolet–visible results, increase in film thickness of IL-templated samples led to significant decrease in the degree of in-plane alignment. We attribute this effect to higher probability of bulk nucleation and reduced influence of template-induced nucleation as the film thickness increases. Interestingly, the in-plane alignment of the reference films did not exhibit such strong film thickness dependence (Fig. 3d), implying that OTS does not serve as a nucleation template and that bulk crystallization dominates in all cases.

Ultraviolet–visible and GIXD studies yielded ensemble-averaged measurement of global alignment. To quantify local alignment, we developed a 2D orientation mapping technique based on TEM electron diffraction^36^. Using this powerful method, we mapped 2D orientation distribution of polymer crystallites over two length scales: 100 × 100 μm^2^ (in a single mesh of the TEM grid) and 1 × 1 mm^2^ (over 100 meshes), at spatial resolutions of 10 and 100 μm, respectively (see Supplementary Method and Supplementary Fig. 14 for details). Typical diffraction patterns from IL-templated DPP2T-TT thin films (10±2 nm) exhibited sharply defined π–π stacking arches of narrow angular intensity distribution, reaffirming the high degree of alignment and molecular ordering induced by dynamic templates. The characteristic d-spacing of the π–π stacking arch is 3.6±0.4 Å (Fig. 3e), consistent with the GIXD measurements (Fig. 3c). By tracking the rotational angle at which the π–π stacking arches appear, the in-plane orientation of crystalline domains can be mapped over scanned areas. Orientation mapping over a single mesh area of 100 × 100 μm^2^ revealed an angular spread of merely 15–20° in most meshes (Supplementary Fig. 14b). Such a narrow orientation distribution was maintained over 100 meshes covering an area of 1 × 1 mm^2^, shown in the histogram of Fig. 3e. We further constructed 2D orientation colour map to visualize the orientation distribution over 1 mm^2^ (Fig. 3e). The colour map uncovered that domains with large orientation deviations (>10°) were concentrated at the two edges of the scanned area, which may have arisen from meniscus instability commonly encountered in MGC processes. Nonetheless, the 2D orientation colour map revealed exceptional in-plane orientational ordering over the majority of the area scanned.

### OFET device performance and charge transport anisotropy

**Figure 4:**
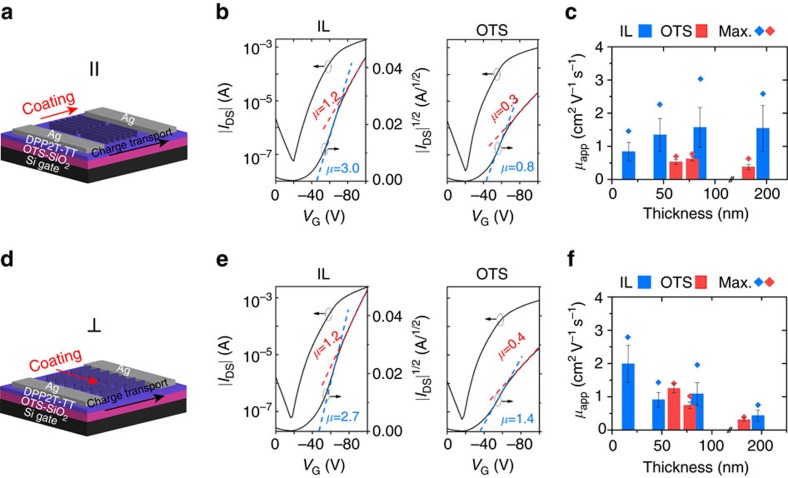
OFET device characterizations comparing IL-templated versus reference devices. (**a**,**d**) Illustration of top contact, bottom gate OFET device configuration with the channel length along (**a**) and transverse (**d**) to the coating direction. (**b**,**e**) Comparison of transfer curves between the best performing IL-templated versus reference devices, measured along (**b**) and transverse (**e**) to the coating direction. IL- and OTS-coated films in panels **b**,**e** share similar *y* axis labels. Due to the presence of the non-ideal ‘kink’ feature, two apparent mobility values are shown, calculated from low *V*_G_ (blue) and high *V*_G_ (red) regions respectively. Corresponding mobility values over the entire range of *V*_G_ are shown in Supplementary Fig. 16c. (**c**,**f**) Comparison of average (column) and maximum (dot) mobilities between IL-templated (blue) and reference devices (red), measured along (**c**) and transverse (**f**) to the coating direction. The mobility values shown are apparent mobilities extracted from the low *V*_G_ region of the transfer curve in the saturation regime (Supplementary Fig. 16a). The corresponding high *V*_G_ mobilities are shown in Supplementary Fig. 16b. The error bars on the average mobilities were s.d. obtained from 20 independent devices.

High degree of polymer alignment enabled by IL templates offers an opportunity to study charge transport anisotropy towards establishing the much-needed structure–property relationships. We are also interested in understanding the interplay between intrachain and interchain charge transport as the degree of alignment is tuned over a wide range. The hole transport properties were measured in bottom gate, top contact OFETs (Fig. 4a,d). To allow direct comparison with the reference devices coated on OTS/SiO_2_ substrates, we transferred the polymer film from the IL/AAO template to the OTS/SiO_2_ substrate following a method described in Supplementary Fig. 7. The residual IL was removed by immersing the transferred film in water. We further verified via X-ray photoelectron spectroscopy (XPS) that there was no detectable IL residual within ∼10 nm near the polymer surface in direct contact with the dielectric layer, which constituted the conductive channel of the transistor devices tested (Supplementary Fig. 15).

Figure 4b,e compare the transfer curves of the best performing IL-templated devices with the reference devices (all transfer curves are summarized in Supplementary Fig. 16a). A typical transfer curve we obtained exhibited a non-ideal downward kink commonly observed in high-performance devices. This phenomenon likely arises from concurrent electron and hole injection and/or field-dependent contact resistance^37^,^38^,^39^. Correspondingly, we calculated apparent mobilities in both low and high gate voltage (*V*_G_) regions (Supplementary Fig. 16a, Fig. 4b,e and Supplementary Table 2). Figure 4c,f and Supplementary Fig. 16b summarize the low *V*_G_ and high *V*_G_ apparent mobilities, respectively, comparing IL templated with the reference devices both parallel and perpendicular to the coating direction. Regardless of the gate voltage range, the apparent mobilities increased by up to fourfold measured parallel to the coating direction (along π-stack) and enhanced by up to twofold perpendicular to the coating direction (along polymer backbone). Although the charge transport at an interface formed by film transfer may not be directly comparable to an interface formed during coating, it is expected that the film transfer process may negatively impact charge transport due to mechanical damage or impurities introduced, as found in previous report^40^. Hence, the extent of charge transport enhancement may be underestimated when comparing IL-templated thin films to reference samples, due to the additional film transfer step needed to prepare the IL-templated devices. We note that the mobility values obtained in this study are not directly comparable to previous reports of DPP2T-TT OFETs^41^, due to the difference in the alkyl chain structure. In addition, no exhaustive effort was spent on optimizing the device performance in this study.

**Figure 5:**
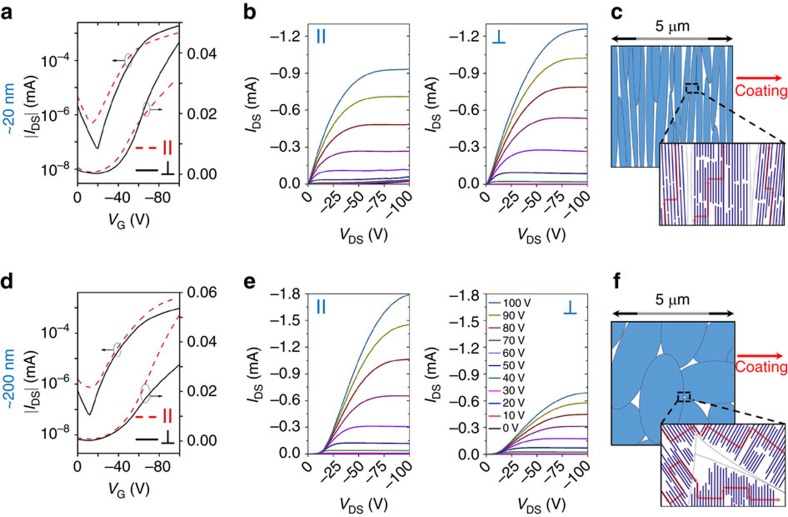
Effect of alignment on charge transport anisotropy in IL-templated films. (**a**,**d**) Transfer and (**b**,**e**) output characteristics of 20 nm thin film of high degree of in-plane alignment (**a**,**b**) versus 200 nm thin film of low degree of in-plane alignment (**d**,**e**), measured along and perpendicular to the coating direction. Corresponding thin film morphology and hole transport path (red arrows) are illustrated in panels **c**,**f**. Corresponding to the transfer curves shown in panel **a**, the peak mobility values are *μ*_par_=1.18 and *μ*_per_=2.72 cm^2^ V^−1^ s^−1^, measured parallel and perpendicular to the coating directions, respectively. For the transfer curves shown in panel **d**, the peak mobility values are *μ*_par_=2.24 and *μ*_per_=0.77 cm^2^ V^−1^ s^−1^, measured parallel and perpendicular to the coating directions, respectively. In other words, dominant charge transport direction is switched for 200 nm films, compared to the case of 20 nm films. In both cases, the peak mobility values were obtained in the low *V*_G_ range. Mobilities calculated in the high *V*_G_ range are shown in Supplementary Fig. 16.

We further investigated the influence of chain alignment on charge transport anisotropy, given that the degree of alignment can be tuned simply by varying the film thickness of IL-templated thin films (Fig. 3). We observed favourable charge transport along the polymer backbone direction in highly aligned 20 nm thin films with *R*_GIXD_=11.8 and *R*_UV–Vis_=5.3 (Fig. 5a–c). In contrast, the preferred charge transport direction flipped to the π–π stacking direction for films of 50–200 nm in thickness, when *R*_GIXD_ decreased to <6.4 and *R*_UV–Vis_<2.5 (Fig. 5d–f; Supplementary Table 2). We attribute this shift in charge transport anisotropy primarily to change in the degree of alignment, but the effect may be complicated by simultaneous variations in domain sizes and therefore grain boundary distributions. Unlike high molecular weight polymers, it has been shown that charge carriers in low molecular weight semicrystalline polymers typically traverse through well-ordered nanocrystalline π-aggregates and move between π-aggregates occasionally through ‘tie-chains’ (Fig. 5f)^10^,^42^. Our results suggest that, even for short polymer chains (*M*_*n*_=20.8 kDa), long-range, high degree of chain alignment can enable favourable charge transport along the conjugated backbone facilitated by occasional interchain hopping through π–π stacking (Fig. 5c).

### Methodology generality

**Figure 6:**
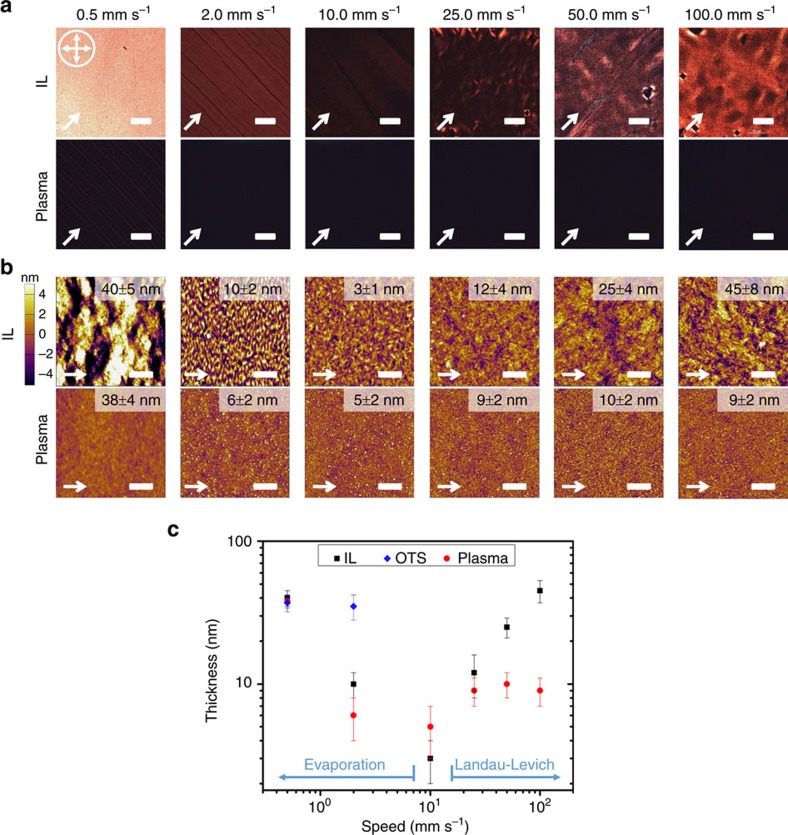
Coating speed dependence of DPP2T-TT film morphology. (**a**) C-POM and (**b**) tapping-mode AFM micrographs comparing IL-templated and reference films coated at a wide range of speed from 0.5 to 100 mm s^−1^. The orientation of the optical microscope cross-polarizers is shown as crossed arrows, and the white arrow indicates the coating direction. All C-POM scale bars are 100 μm and all scale bars in the AFM height images are 1 μm. Film thickness is denoted on the upper right corner in all images. (**c**) DPP2T-TT film thickness as a function of coating speed. Evaporation and Landau–Levich regimes can be identified from films coated on IL template. Reference samples did not follow the same trend predicted by theory.

To demonstrate the generality of our concept, we show that the IL-based dynamic template is effective over a wide range of coating speed spanning over two orders of magnitude from 0.5 to 100 mm s^−1^; we further validate the dynamic templating approach using a second donor–acceptor polymer system. For the coating speed study, we employed plasma-treated SiO_2_/Si substrate as a reference in addition to OTS-treated substrate, because inadequate wetting prevents film deposition on OTS substrate at elevated coating speeds (Supplementary Fig. 17). Across the entire speed range tested, IL-templated thin films exhibited significantly higher brightness under cross-polarizers and larger crystallite sizes revealed by AFM compared to films coated on plasma-treated substrates (Fig. 6a,b), as well as those on OTS substrates when the films could be deposited (Supplementary Fig. 17). Intriguingly, at coating speed as high as 100 mm s^−1^, we still obtained a highly crystalline film on IL template, verifying that the dynamic template indeed significantly expedited polymer crystallization to alleviate the timescale mismatch during rapid coating.

For IL-templated films, the morphology (Fig. 6a,b) and film thickness (Fig. 6c) variations as a function of coating speed are expected and consistent with the previously published solution coating model^43^. The work by Le Berre *et al*.^43^ laid out two coating regimes: the evaporation regime and the Landau–Levich regime. In the evaporation regime (coating speed <10 mm s^−1^ in our case; Fig. 6c), solvent evaporation induces solute supersaturation at the meniscus, resulting in nucleation at the contact line and causing the crystal growth to follow the receding meniscus. This regime often yields aligned crystalline domains provided that the crystal growth is sufficiently rapid, which is what we observed (Fig. 6a,b). We also observed that the film thickness decreases with coating speed, a trend consistent with the model prediction for the evaporation regime. In the Landau–Levich regime (coating speed >10 mm s^−1^ in our case; Fig. 6c), viscous drag-out dominates over solvent evaporation. In this regime, the liquid film drag-out is followed by uniform solvent evaporation, initiating sporadic nucleation across the entire liquid film to yield isotropically oriented domains as we observed (Fig. 6a,b). The film thickness increases with the coating speed in this regime, which we also observed. On the other hand, films coated on the two reference substrates did not exhibit a clear regime change, neither did the film thickness follow the trend predicted by theory. Such deviation may be caused by insufficient wetting on OTS and plasma-treated substrates, which altered viscous drag-out, and/or that the polymer crystallization rate was too slow to trace the receding meniscus for inducing domain alignment. The stark contrast between IL-templated and the reference films across the entire range of speed validated our design concept that the IL dynamic template expedites polymer crystallization to alleviate its kinetics mismatch with rapid coating. The IL template further enhances wetting with the ink solution to promote film formation.

**Figure 7:**
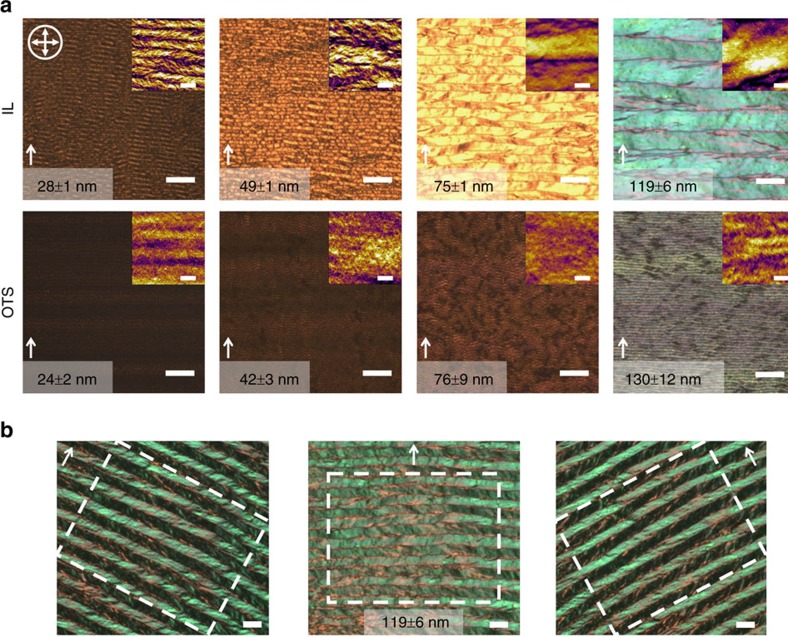
Effect of dynamic templates on PII-2T thin film morphology. (**a**) Polarized optical microscopy images of IL-templated PII-2T thin films compared with reference thin films deposited on OTS. Crossed-arrows denote crossed polarizers orientation and white arrows show the coating direction. All scale bars in the optical images are 10 μm. Inset: Tapping-mode AFM height images of corresponding PII-2T thin films with 1 μm scale bars. (**b**) Polarized optical microscopy images of IL-templated PII-2T thin films exhibiting twinned crystalline domains. All scale bars are 10 μm.

To further evaluate the generality of our methodology, we tested another conjugated polymer, PII-2T, with the same alkyl chain design but different conjugated backbone. MD simulations suggested even stronger interactions between the PII-2T backbone and [EMIM][TFSI], judged from higher maximum excess probability of 9 compared to 6 for DPP2T-TT (Fig. 1c). Similar to the case of DPP2T-TT, the excess probability of PII-2T was also diminished when IL molecules were ‘frozen’ in place, validating that the dynamic nature of the IL surface was responsible for enhanced interactions with the conjugated backbone. Correspondingly, we observed even more dramatic improvement in PII-2T morphology comparing IL-templated to reference thin films (Fig. 7). Across a wide range of film thicknesses tested, application of dynamic template led to significantly larger domain sizes and markedly higher birefringence (Fig. 7a). Most strikingly, we observed twinned domains exceeding 10 μm in width and up to centimetres long, which resembles the morphology characteristics of highly crystalline small molecules rarely found in conjugated polymer thin films (Fig. 7b). The two examples presented in this work suggest that the observed dynamic-template-directed polymer crystallization may be a general phenomenon and further point to the tantalizing possibility to rationally design dynamic templates through evaluating the polymer excess probability distribution.

## Discussion

In summary, we demonstrated the concept of dynamic templating for enhancing polymer crystallization and assembly during MGC using two conjugated polymers DPP2T-TT and PII-2T as model systems. The model dynamic templates were constructed from IL [EMIM][TFSI] hosted in nanoporous AAO. Application of dynamic templates during MGC led to highly aligned, highly ordered polymer thin films over a large area (>1 cm^2^). The aligned films exhibited dichroic ratio as high as 10.1 by ultraviolet–visible and 22.6 by GIXD, which were among the highest values reported for conjugated polymers. Orientation mapping via TEM electron diffraction further revealed that the angular spread of polymer crystalline domains was as narrow as ±10°. Improved alignment and crystallinity in dynamic-template-directed polymer thin films led to enhanced charge carrier mobility along both the polymer backbone and the π–π stacking direction. Interestingly, we observed favourable charge transport along the polymer backbone but only when the polymer chains were highly aligned.

We propose the following mechanism for dynamic-template-directed assembly during MGC based on MD simulation results. Owing to the dynamic nature and reconfigurability of the IL surface, the ion–π interactions between the IL and the polymer backbone were dramatically enhanced, resulting in the formation of a highly enriched polymer layer at the IL template. The polymer enrichment lowered the nucleation barrier and expedited polymer crystallization to alleviate the timescale mismatch during rapid solution coating. As a result, polymer crystal growth was sufficiently rapid to follow the receding meniscus, wherein the unidirectional capillary flow guided the film growth along the fastest growth axis—the π–π stacking direction. The synergy between dynamic-templated-induced nucleation and unidirectional-flow-guided crystal growth led to the formation of highly aligned, highly crystalline polymer thin films. We believe that our strategy of dynamic-template-directed assembly can be applied to a wide range of molecular systems to realize high degree of morphology control otherwise challenging to achieve.

## Methods

### ^1^H NMR spectroscopy

Solution-state proton NMR spectra of the DPP2T-TT solution in the absence and presence of [EMIM][TFSI] were collected on a Varian Inova 600 MHz NMR spectrometer. We prepared the mixture of 1:100 molar ratio of DPP2T-TT repeating unit (∼1 mM) and IL (∼100 mM) in deuterated chloroform with 1% tetramethylsilane by dissolving DPP2T-TT in chloroform and then adding [EMIM][TFSI] to the mixture (solutions were stirred overnight). The tetramethylsilane at 0 p.p.m. was used to reference all proton spectra.

### MD simulations

The starting structures of [EMIM]^+^, [TFSI]^−^, chloroform, monomers and dimers were drawn using Maestro^44^ and optimized by its built-in function. The cubic simulation box of 100 Å in length containing an IL layer and a polymer solution layer were generated using Packmol^45^. All the simulations were set up using the AMBERTools14 and performed with the AMBER14 software^46^ using the general AMBER force field. All simulations were performed in NPT ensemble (1 atm, 298 K) with periodic boundary conditions. The integration step was 2 fs. Cartesian coordinate restraints were applied to restraint the atom positions in the static IL simulations. Simulations were performed on the Blue Waters petascale computing facility. For more details, please see Supplementary Method.

### Meniscus-guided coating

We infiltrated nanoporous substrate of AAO membrane with 200 nm pore size and 13 mm diameter (purchased from Sigma-Aldrich) with [EMIM][TFSI]≥98% (purchased from Solvionic) to form a semisolid state IL/AAO hybrid supported by glass substrate. The DPP2T-TT (*M*_*n*_=20,052 g mol^−1^, *M*_W_=54,791 g mol^−1^) and PII-2T (*M*_*n*_=210,805 g mol^−1^, *M*_W_=722,849 g mol^−1^) synthesis procedures are summarized in Supplementary Method. In both cases, the solvent used was chloroform (99.8% ACS-grade purchased from Sigma-Aldrich). Film thickness was varied by changing the solution concentration between 1 and 20 mg ml^−1^ as opposed to varying the substrate temperature or the coating speed, so that the evaporation rate can be maintained constant. During MGC, the ink solution was sandwiched between a slightly tilted, OTS-functionalized blade and the substrate. The tilting angle of the blade was 8°. The gap between the substrate and the blade was set as 100 μm. During coating, the blade was linearly translated at a coating speed of 0.5 mm s^−1^, and the substrate temperature was set at 25 °C. After the films were coated on IL/AAO hybrid substrates, they were transferred to OTS-functionalized silicon wafer with 300 nm thermally grown SiO_2_ by simply bringing the substrate in contact with the film. The transferred films were subsequently immersed in water for at least 3 h to remove the IL residual. All SiO_2_-Si substrates used in this work were treated with OTS to minimize interfacial charge traps. OTS treatment was accomplished by immersing the plasma-treated, precleaned wafer in a trichloroethylene solution of OTS (0.2 vol%) at room temperature for 20 min. The treated wafers were then rinsed and baked at 120 °C for 20 min.

### Film morphology characterizations

For all measurement, DPP2T-TT was deposited on OTS-treated silicon substrate to serve as reference samples. Polymers coated on IL/AAO hybrid substrates were transferred to OTS-treated substrates or TEM grid before morphology characterizations. Fabricated polymer films were first visualized using a Nikon Ci-POL optical microscope to observe birefringence under cross-polarized light. AFM measurements were performed under the tapping mode using an Asylum Research Cypher instrument, from which the mesoscale morphologies were compared and the film thickness was measured. Polarized ultraviolet–visible absorption spectra were recorded at room temperature on an Agilent Cary 60 ultraviolet–visible spectrophotometer, with the incident light polarized vertically by a broadband thin film polarizer. The scans were taken from 400 to 1000, nm. GIXD measurements were performed at beamline 8-ID-E at the Argonne National Laboratory, with an X-ray wavelength of 1.6868 Å (*E*_beam_=7.35 keV), at a 208 mm sample-to-detector distance. A 2D Pilatus 1M detector was used for data collection. Samples were scanned for 10 s in a Helium environment at an incident angle of 0.2°. Data analysis was performed using the software GIXSGUI^47^. The edge-on π–π stacking peak and the lamella stacking peak were obtained from a sector cut between −88°<*χ*<−83° and −10°<χ<−5°, respectively, from the geometrically corrected image. Careful multi-peak fitting was performed to deconvolute the π–π stacking peak from the amorphous ring, SiO_2_ scattering and the OTS peak. The π–π stacking peak was fitted with a Gaussian function to obtain the peak position and peak area for determining π–π stacking distance and dichroic ratio. The peak area was further normalized by the irradiated volume to allow comparison across samples. Orientation mapping was carried out by selected-area electron diffraction using a JEOL 2100 Cryo TEM at accelerating voltage of 200 kV and a camera length (*L*) of 50 cm. The samples for electron microscopy were prepared by transferring the films from IL/AAO onto a copper grid with carbon support. Diffraction patterns were recorded using a 2*k* × 2*k* CCD camera at a binning of 4 and 1 s exposure time. The selected area has a diameter of ∼0.8 μm. We scanned the beam across the thin film by manually changing the sample position.

### X-ray photoelectron spectroscopy

The XPS data was obtained using a Kratos Axis Ultra XPS system (Krato Anaytical Ltd.) under high vacuum (1 × 10^−9^ torr) using a monochromatic Al-K_α_ X-ray source (14 kV, 10 mA). High-resolution XPS spectra were obtained at a constant pass energy of 40 eV and a step size of 0.1 eV and an exposure time of 400 ms. Peak analysis and deconvolution was performed with the CasaXPS software.

### Device fabrication and electrical characterization

The DPP2T-TT OFETs with bottom gate, top-contact configuration was fabricated on highly n-doped Si (gate) with an OTS-functionalized 300 nm SiO_2_ layer (dielectric). Silver source and drain electrodes of 40 nm thick were thermally evaporated onto the polymer films through a shadow mask. The channel length (*L*) was 52 μm and channel width (*W*) was 4.4 mm. All electrical measurements were performed in a nitrogen environment using a Keysight B1500A semiconductor parameter analyser. The field-effect mobilities were calculated from the saturation region of transfer curves by the equation, 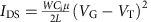, where *I*_DS_ is the drain-source current, *C*_i_ is the capacitance of the dielectric (11 nF cm^−2^ for OTS-treated 300 nm SiO_2_ dielectric), *V*_G_ is the gate voltage, *μ* is the apparant mobility and *V*_T_ is the threshold voltage. *W* and *L* are the channel width and length of the organic semiconductor, respectively. Average data were calculated from analysis of 20 independent devices.

### Data availability

All data is available from the authors upon reasonable request.

## Additional information

**How to cite this article:** Mohammadi, E. *et al*. Dynamic-template-directed multiscale assembly for large-area coating of highly-aligned conjugated polymer thin films. *Nat. Commun.* **8,** 16070 doi: 10.1038/ncomms16070 (2017).

**Publisher’s note:** Springer Nature remains neutral with regard to jurisdictional claims in published maps and institutional affiliations.

## Supplementary Material

Supplementary Information
