# Supplementary material for: Dynamic-template-directed multiscale assembly for large-area coating of highly-aligned conjugated polymer thin films
Source: Nat Commun. 2017 Jul 13;8:16070. doi: 10.1038/ncomms16070 (PMC5511351; doi:10.1038/ncomms16070)
Supplement: Supplementary Information [file ncomms16070-s1.pdf]

File Name: Supplementary Information
Description: Supplementary Figures, Supplementary Tables, Supplementary Note, Supplementary Methods and Supplementary References

Supplementary Note

Stability of Dynamic Template During Solution Coating. To ensure the compatibility of the AAO/IL hybrid dynamic template with the solution coating process, the porous AAO membrane must be stably infiltrated by IL (Configuration 1 in Supplementary Figure 1). Otherwise, the polymer ink solution may replace the IL during coating (Configuration 2 in Supplementary Figure 1). In other words, the IL-infiltrated AAO covered by the ink solution represents a lower free energy state than the ink-infiltrated AAO covered by the IL. Let E_1 and E_2 represent the total interfacial free energies of configurations 1 and 2 respectively[1]:

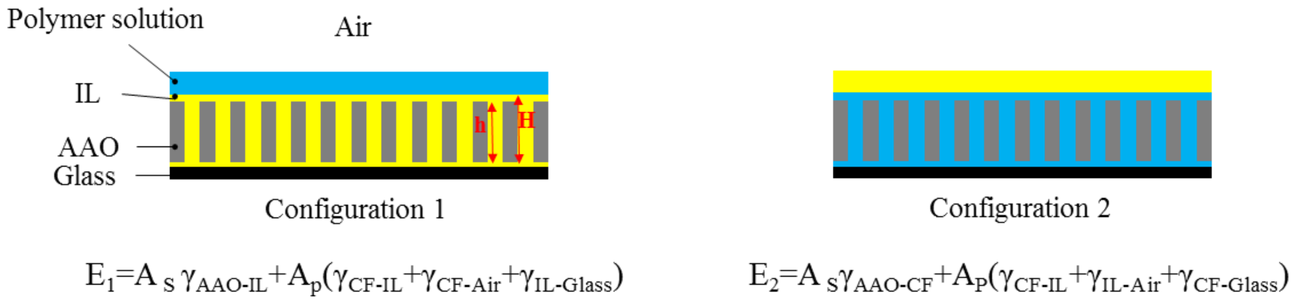

Supplementary Figure 1. Configurations of the hybrid templates with and without the ink replacing the IL. Maintaining configuration 1 is necessary for coating on the dynamic template. However, if this configuration is unstable, the ink will replace the IL and configuration 2 will prevail.

Herein, we assume that IL forms a wetting layer on top of the AAO membrane (i.e., $H > h$ shown in Configuration 1, and the surface tension of the polymer solution is comparable to that of the solvent given the low polymer concentration (~1 w%) and low molecular weight (Mn=20.8 kg.mol^{-1}). The energy for each configuration can be expressed as,

$$E_1 = A_S \gamma_{AAO-IL} + A_P(\gamma_{CF-IL} + \gamma_{CF-Air} + \gamma_{IL-Glass}) \quad (1)$$

$$E_2 = A_S \gamma_{AAO-CF} + A_P(\gamma_{CF-IL} + \gamma_{IL-Air} + \gamma_{CF-Glass}) \quad (2)$$

Wherein A_S and A_P denote the total membrane surface area and the projected area respectively. Consequently, the energy difference between these two configurations (ΔE) can be calculated,

$$\Delta E = A_S(\gamma_{AAO-CF} - \gamma_{AAO-IL}) + A_P((\gamma_{IL-Air} - \gamma_{CF-Air}) + (\gamma_{CF-Glass} - \gamma_{IL-Glass})) \quad (3)$$

We define aspect ratio R, as A_S/A_P, and simplify Supplementary Equation 3 as,

$$\frac{\Delta E}{A_P} = R(\gamma_{AAO-CF} - \gamma_{AAO-IL}) + (\gamma_{IL-Air} - \gamma_{CF-Air}) + (\gamma_{CF-Glass} - \gamma_{IL-Glass}) \quad (4)$$

Configuration 2 should be always at a higher energy state than configuration 1, or $\Delta E=E_2-E_1>0$, to form a stable template for coating. To further simplify Supplementary Equation 4, we used Young's equation and measured the contact angle of [EMIM][TFSI] and chloroform on polished sapphire wafer (purchased from University Wafer, Inc.) and glass slide. The sapphire wafer is used to model the surface chemistry of alumina surface in AAO membrane given the same composition,

$$\gamma_{AAO-IL} = \gamma_{AAO-Air} - \gamma_{IL-Air} cos\theta_{IL-AAO} \quad (5)$$

$$\gamma_{AAO-CF} = \gamma_{AAO-Air} - \gamma_{CF-Air} cos\theta_{CF-AAO} \quad (6)$$

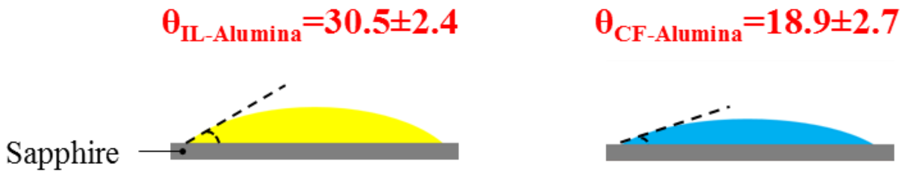

Supplementary Figure 2. [EMIM][TFSI] and chloroform contact angles on sapphire.

$$\gamma_{IL-Glass} = \gamma_{Glass-Air} - \gamma_{IL-Air} cos\theta_{IL-Glass} \quad (7)$$

$$\gamma_{CF-Glass} = \gamma_{Glass-Air} - \gamma_{CF-Glass} cos\theta_{CF-Glass} \quad (8)$$

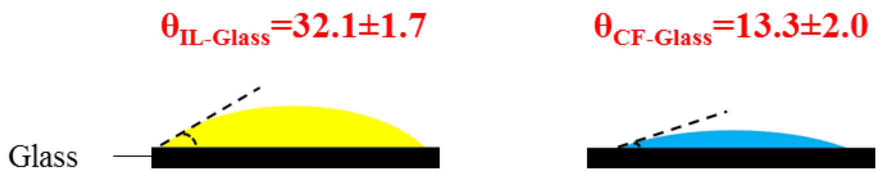

Supplementary Figure 3. [EMIM][TFSI] and chloroform contact angles on glass.

By substituting Supplementary Equations 5-8 into Supplementary Equation 4 and simplifying the equation, we have,

$$\frac{\Delta E}{A_P} = R(\gamma_{IL-Air}cos\theta_{IL-AAO} - \gamma_{CF-Air}cos\theta_{CF-AAO}) + (\gamma_{IL-Air} - \gamma_{CF-Air}) + (\gamma_{IL-Air}cos\theta_{IL-Glass} - \gamma_{CF-Air}cos\theta_{CF-Glass}) \quad (9)$$

A_S for AAO structure can be estimated as,

$$A_S = A_{Wall} + 2A_{Surface} = \left(A_P \times d_{pore} \times 2\pi rl\right) + 2\left(A_P - A_P \times d_{pore} \times \pi r^2\right)$$

$$A_S = 2A_P\ (1 + d_{pore}\pi r(l - r)) \quad (10)$$

Where r is the pore radius (0.1 μm), l is the membrane thickness (60 μm) and d_{pore} is the pore density (10^{13} m^{-2}). Now we can calculate R,

$$R = \frac{A_S}{A_P} = 2\left(1 + d_{pore}\pi r(l - r)\right)$$

$$= 2\left(1 + \frac{10^{13}}{m^2} \times \pi \times 0.1 \times 10^{-6} m \times (60 - 0.1) \times 10^{-6} m\right) = 378 \qquad (11)$$

Surface tension of pure [EMIM][TFSI] and chloroform are measured to be 36.2±0.7 mJ/m^2 and 27.5±1.7 mJ/m^2 respectively, measured by the pendant drop method using Rame-Hart Model 250 Standard Goniometer. Thus, $\Delta E/A_P$ will be,

$$\frac{\Delta E}{A_P} = 378\big(36.2\ cos(30.5) – 27.5cos(18.9)\big) + (36.2 - 27.5)$$

$$+\ \big(36.2\ cos(32.1) – 27.5cos(13.3)\big) = (378(5.2) + 8.7 + 3.9)\frac{mJ}{m2}$$

$$= 1.97\frac{J}{m2} \tag{12}$$

These calculations prove the stability of AAO/IL hybrid implemented in this work, and establish a model for predicting the hybrid template stability, which can be used for other IL and solvent pairs.

Supplementary Methods

Molecular Dynamics Simulations. Molecular dynamics simulations on monomeric and dimeric backbones of DPP2T-TT and PII-2T were performed to investigate the effect of dynamics on the surface adsorption of conjugated polymers onto our proposed ionic liquid (IL), [EMIM][TFSI]. Simulation on a DPP2T-TT 18-unit polymer chain (average length of the actual polymer used) was also performed to investigate the conformation of the actual polymer chains in chloroform solvent. This is to confirm that the polymer backbone is fully exposed to IL, in order to justify the removal of alkyl chains in the oligomer simulation. Lastly, simulation on pure chloroform molecules and ILs was performed to calculate the buffer distributions of IL molecules, in order to compare with the IL distribution around the polymers. Simulation details are summarized in the table below:

Supplementary Table 1. Summary of molecular dynamics simulations details.

System		ILs	IL pairs	$CHCl_3$	Simulation box (Å)	IL layer (Å)	Simulation time (μs)
DPP2T-TT	five monomer backbones	dynamic	550	5600	100*100*100	25	2.0
	dimer backbone	dynamic	550	5600	100*100*100	25	2.0
	dimer backbone	static	550	5600	100*100*100	25	3.6
	18-unit polymer chain	/	/	26630	348*91*130	/	0.07

PII2T	five monomer backbones	dynamic	550	5600	100*100*100	25	2.0
	dimer backbone	dynamic	550	5600	100*100*100	25	2.0
	dimer backbone	static	550	5600	100*100*100	25	3.6
pure chloroform and ILs		dynamic	550	3600	90*90*90	30	0.1

Initial Setup of Monomer/Dimer Simulations. The starting structures of $[EMIM]^+$, $[TFSI]^-$, $CHCl_3$, monomers and dimers were drawn using Maestro[2] and optimized by its built-in Geometry Cleanup function. The non-interacting alkyl chains of the monomers and dimers were removed to capture the molecular interactions between the polymer backbones and the ILs. Cubic simulation boxes of 100 Å in length containing 550 pairs of [EMIM][TFSI], 5600 $CHCl_3$ molecules, and 5 monomers or a single dimer were generated using the Packmol[3] at close to the experimental densities. The number of IL ion pairs was chosen such that the long-range interactions and 'bulk liquid' effects can be fully captured according to prior theoretical studies on ILs[4]. The monomers and dimers were randomly distributed in the $CHCl_3$ solvent. The simulations were launched from multiple initial configurations in parallel.

Initial setup of DPP2T-TT 18-unit polymer chain simulation. The initial polymer chain structure model was generated by repeating the monomer structure for 18 times in Maestro[2]. After the structure optimization, the polymer chain was then solvated with 26630 chloroform molecules in tleap. The simulation box size was 348*91*130 Å and there are at least 12 Å between the polymer chain atoms and the boundary of simulation box. A single simulation was launched from the solvated structure.

Initial setup of pure chloroform simulation. Cubic simulation box of 90 Å in length containing 550 pairs of [EMIM][TFSI], 3600 chloroform molecules was used as the starting configuration for simulation.

All the simulations were set up using the AMBERTools14 and performed with AMBER14 software[5] using the general AMBER force field (GAFF). All the partial charges were derived using the AM1/BCC method. The same partial charges from the monomer DPP2T-TT were used for each unit of the DPP2T-TT polymer chain. Previous studies have shown that the GAFF can accurately predict transport and thermodynamic properties of ILs[6]. The system energy was first minimized with 2000 steps of steepest-descent method and then with 8000 steps of conjugated-gradient method. Subsequently, the systems were slowly heated from 0 to 10 K in NVT and NPT ensemble for 1 ns, respectively. Then the systems were heated from 10 K to 298 K in NPT ensemble for 2 ns. Cartesian coordinate restraints were applied on all heavy atoms during the minimization and heating stages with a force constant of 20 kcal/mol. Before the production runs, the systems were equilibrated without any restraints in NPT ensemble (1 atm, 298 K) for 1 ns.

All the simulations were performed in NPT ensemble (1 atm, 298 K) with periodic boundary conditions. Particle-mesh Ewald[7] method was used to treat the electrostatic interactions with a 10 Å cutoff distance. The SHAKE algorithm[8] was applied to constrain the length of covalent bonds involved hydrogen atoms to their equilibrium values. The integration step was 2 fs. Berendsen thermos-barostat[9] with a damping time constant of 2 ps was used to control the temperature and pressure of the ensembles. To study the effects of ILs dynamics on the surface adsorption of polymers onto ILs, production runs with and without position restraints on the ILs were performed for the dimeric backbones of DPP2T-TT and PII2T-TT simulations. In the case of static ILs,

position restraints with a force constant of 10 kcal/mol were applied on all heavy atoms of ILs. Simulations were performed on the Blue Waters petascale computing facility.

Distribution Probability Analysis. For the monomer/dimer simulations, all the production trajectories were processed with CPPTRAJ[10] so that the ILs layers were positioned at the center of simulation boxes. The distance r was defined as the center of mass distances between the polymer backbones and ILs in the direction orthogonal to the initial $CHCl_3$/ILs interface. The normalized distribution probability of polymers p(r) were calculated with MDTraj 1.7.2 package[11]. Excess probability was defined to directly compare the probability of the polymer at the ILs interface and the bulk. The bulk excess probability is 1.

$$Excess\ probability = \frac{Normalized\ probability\ p(r)}{Bulk\ normalized\ probability\ p(bulk)} \qquad (13)$$

Radius distribution function analysis. The radius distribution functions $g(r)$ of cation and anion around the function groups of DPP2T-TT dimeric backbone were calculated from the dynamic ILs simulations. The radius distribution function is defined as

$$g(r) = \frac{n}{\rho * 2\pi r^2 dr} \qquad (14)$$

where r is the distance to the backbone atom, *n* is the number of cations or anions within *dr* region from *r* to *r*+*dr*, ρ is the bulk ion concentrations, $2\pi r^2$ is the area of hemisphere with a radius of *r* (only the hemisphere in the IL layer was filled with ions). It should be noted that the ILs interface fluctuates in the simulations so that $g(r)$ doesn't converge to 1. In this study, we calculated the radius distribution functions of cation and anion near the carbonyl and the thiophene function groups. The distances were calculated from the charge centers of ions to the oxygen and sulfur atom in each group, respectively. The data used for these calculations were taken from 3000

snapshots of the raw trajectories where the backbones were absorbed on the ILs interface. The buffer distributions of cations and anions were calculated by averaging the radius distribution functions from 20 random points near the IL interface.

DPP2T-TT polymer chain simulation analysis. The backbone solvent accessible surface areas were calculated using MDTraj[11] 1.7.2 package. During the entire simulation, the solvent accessible surface area (SASA) of the conjugated backbone fluctuates within the range of 60 ± 3 nm^2. The small variance in backbone SASA indicates the convergence of our simulations.

TEM electron diffraction-based two-dimensional orientation mapping procedure. We recorded electron diffraction patterns to cover 100 meshes (10 by 10) on a 3mm diameter TEM grid with 100 μm×100 μm mesh size using a selected beam depicted in Figure S7-a. We translated the sample and acquired the diffraction patterns (DPs) in a step-by-step manner to scan over the entire designated area on the thin film. Within each mesh, the step size was 10 μm to yield 100 scans per mesh. A total of 100 meshes were scanned to cover an area of 1 mm^2 (Fig. S7). Within each mesh of 0.01 mm^2, we plotted the 2D orientation distribution as a histogram of 100 scans, scattered in terms of the rotation angle of the π-π stacking peak relative to an arbitrarily defined fixed axis. The histograms from five representative meshes are shown in Figure S7-b. For plotting the 2D orientation distribution over 100 meshes of 1 mm^2, we used the average rotation angle of the π-π stacking peak from each mesh, and constructed the histogram shown in Figure 3e. The average rotation angle was normalized as zero in this plot.

Constructing the 2D orientation color map shown in Figure 3e requires grouping similar diffraction patterns. We applied image cross-correlation analysis to cluster the DPs from each mesh[12]. Since the brightness of the DPs can vary from mesh to mesh, the similarity of images is compared using normalized cross-correlation factor making the analysis independent of the image intensity:

$$\gamma = \frac{\sum_{x,y}\{[I_A(x,y) - \overline{I_A}][I_B(x,y) - \overline{I_B}]\}}{(\{\sum_{x,y}[I_A(x,y) - \overline{I_A}]^2\}\{\sum_{x,y}[I_B(x,y) - \overline{I_B}]^2\})^{1/2}} \qquad (15)$$

where $I_A(x, y)$ and $I_B(x, y)$ are the intensities of a pixel (x, y) in images A and B, respectively, and $\overline{I_A}$ and $\overline{I_B}$ are the mean intensities of images A and B, respectively[13]. The construction of the 2D orientation color map requires applying a correlation factor (γ) threshold for grouping similar images (similar orientation judged from the π-π stacking rotational angle) into distinct clusters.

The cross-correlation threshold was chosen by trial and error and it was set at >95.5% for binning γ of similar values. We estimate that this threshold value can distinguish DPs with angular spread within 4 degrees of the bin average. Our orientation mapping method should be broadly applicable for quantifying local orientation in-plane in a wide range of crystalline and semi-crystalline thin film systems.

Interestingly, we observed single-crystal-like hexagonal diffraction patterns with very sharp peaks occasionally during mapping (Fig. S-7c). Similar patterns were reported for TA-PPE conjugated polymer nanowire crystals (a derivative of poly(para-phenylene ethynylene) with thioacetate end groups)[14]. This may be due to the broad distribution in molecular weight of the DPP2T-TT polymer synthesized.

Polymer synthesis. The conjugated polymer DPP2T-TT was synthesized following a previously published procedure[15]. 3,6-bis(5-bromothiophen-2-yl)-2,5-bis(4-decyltetradecyl) pyrrolo[3,4-*c*]pyrrole-1,4(2*H*,5*H*)-dione (300.0 mg, 265.2 mmol) and 2,5-bis (trimethylstannyl)thieno[3,2-*b*]thiophene (123.5 mg, 265.2 mmol) were dissolved in 20 mL of toluene in a 35 mL microwave reaction vessel. The solution was purged with nitrogen for 15 minutes, before tris(*o*-tolyl)phosphine (3 mg) and tris (dibenzylideneacetone)dipalladium(0) (6 mg) were added. The vessel was sealed with a snap cap and quickly transferred to a CEM Discover Microwave Reactor. Reaction conditions were listed as follows: Power cycling mode; Power, 300 W; Power cycles, 100; Temperature, 120 – 150 °C; Heating, 120 s; Cooling, 30 s; Pressure, 150 psi; Stirring, high. After the reaction was complete, the polymer was collected by precipitation into methanol. The product was dissolved in 50 mL of chloroform and palladium was removed with 30 mg of *N*,*N*-diethylphenylazothioformamide at 50 °C for 30 minutes. The solution was precipitated into methanol and the solid was dried under 60 °C over high vacuum. The PII-2T synthesis procedure was detailed in a previous publication[16].

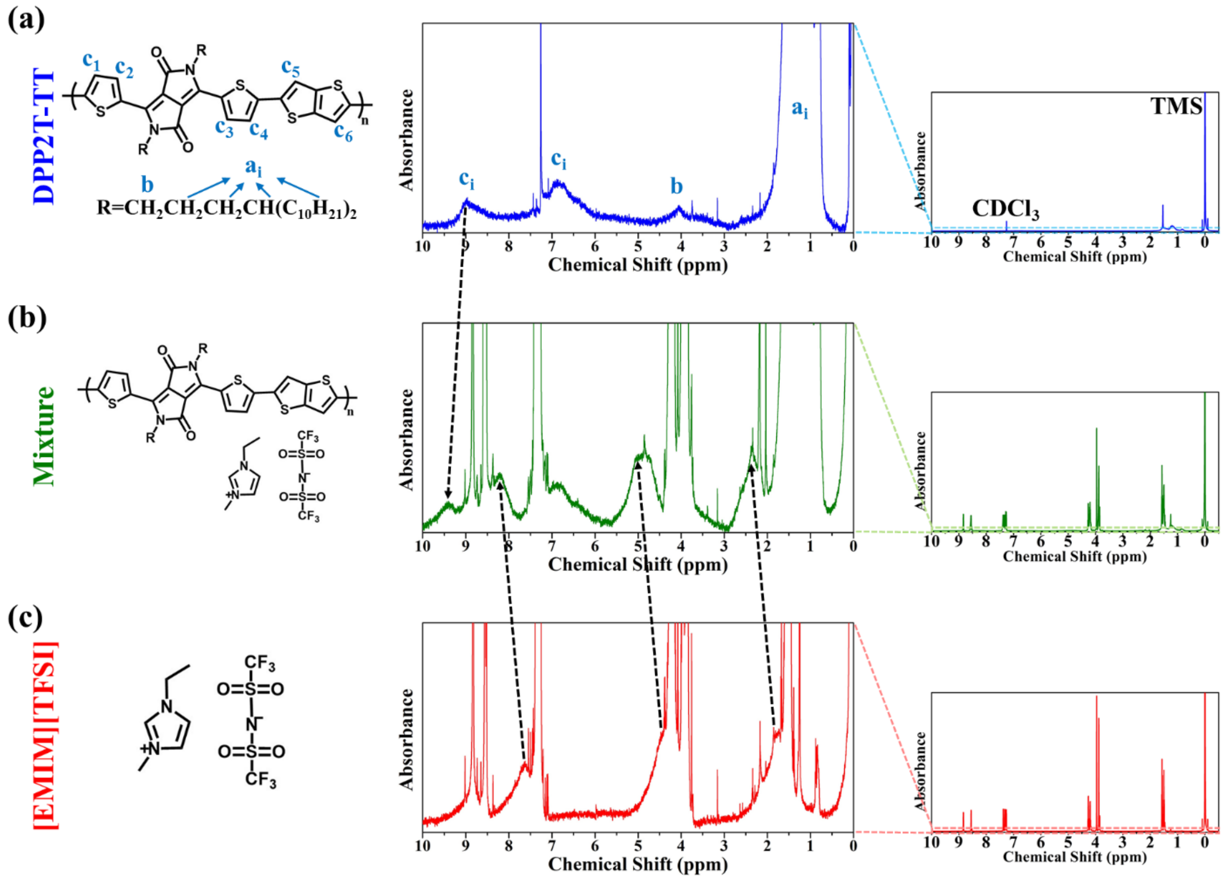

Supplementary Figure 4. ^{1}H NMR spectrum of DPP2T-TT ([EMIM][TFSI]) solution with and without [EMIM][TFSI] (DPP2T-TT). (a) DPP2T-TT (~1 mM), (b) DPP2T-TT:[EMIM][TFSI] (1:100 molar ratio) and (c) [EMIM][TFSI] (~100 mM) in deuterated chloroform as the solvent. Down-field chemical shift change observed in DPP2T-TT (9.0 to 9.4 ppm) spectrum by adding [EMIM][TFSI] to the solution is ascribed to changes in the polymer backbone protons (marked as c_i in the represented molecular structure) environment due to the presence [EMIM][TFSI] ions. Correspondingly, [EMIM][TFSI] spectrum showed significant and consistent shifts in three peaks positions in the polymer-IL mixture compared to the solution in absence of DPP2T-TT. Polymer alkyl chain protons (a_i and b) didn't experience any noticeable chemical shift by adding [EMIM][TFSI] to the solution. The chemical shifts of the backbone protons indicate different chemical environment around the DPP2T-TT conjugated core in the presence of IL while

the alkyl chain interactions are not influenced by the presence of ions. Therefore, [EMIM][TFSI] interactions with polymer molecules are mainly through the π-conjugated system which increases the electron-withdrawing effect near the backbone to result in the deshielding effect verified by the down-field shift[17-19].

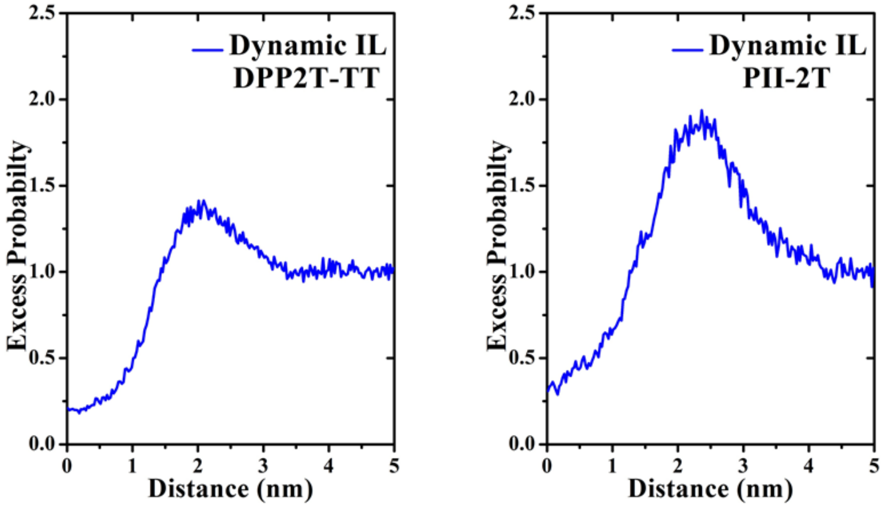

Supplementary Figure 5. Molecular dynamics simulation analysis. Excess distribution probability curves with respect to the IL surface for monomeric DPP2-TT and PII-2T in the case of dynamic IL.

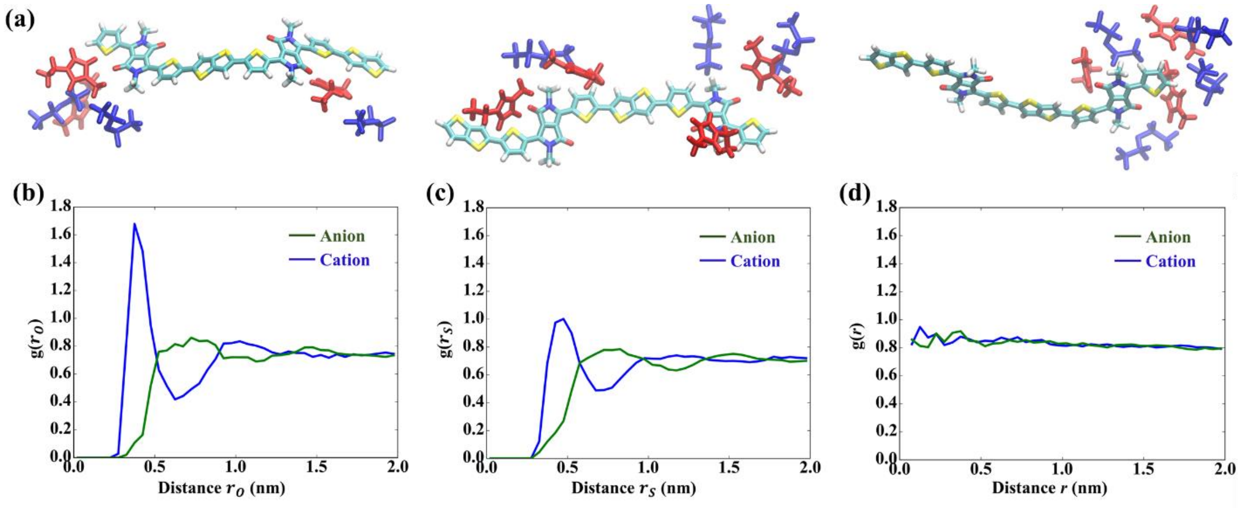

Supplementary Figure 6. [EMIM][TFSI] ion distribution in chloroform solution with respect to DPP2T-TT backbone. (a) Snapshots of [EMIM][TFSI] ions surrounding the DPP2T-TT backbone from the static IL simulations contributing to the small peaks in the excess distribution probability curve near the surface. The cations and anions are all within 5 Å from the backbone and shown in red and blue respectively. Radial distribution functions of cations and anions near (b) the oxygen atoms in the carbonyl function groups and (c) the sulfur atoms in the thiophene functional groups of the backbone in the presence of DPP2T-TT. (d) Radius distribution functions of cations and anions in the pure chloroform without DPP2T-TT present.

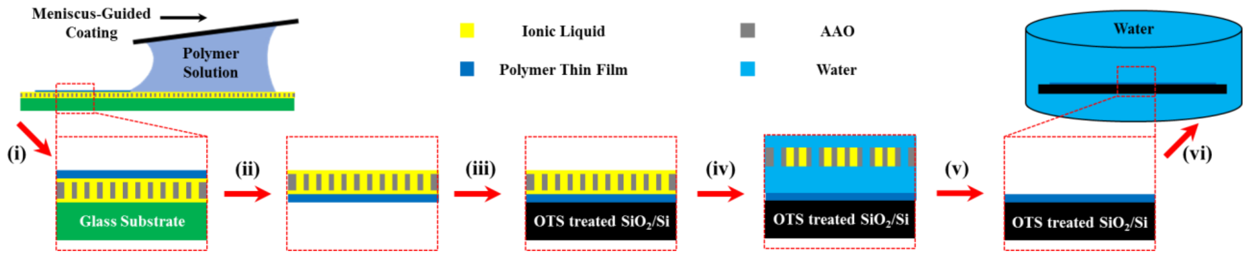

Supplementary Figure 7. Schematic illustration of the polymer thin film coating and transfer process to OTS treated SiO_2/Si substrate. (i) Coating the polymer thin film on the IL-dynamic template via solution coating, (ii) removing the AAO/IL/polymer thin film from glass substrate using a Micro Fine Tip Tweezer and flipping it 180°, (iii) transferring the AAO/IL/polymer thin film to the OTS treated SiO_2/Si substrate by simply contacting it with the OTS treated SiO2/Si substrate (no additional force is needed), (iv) adding sufficient amount of water to detach the AAO/IL from the substrate by dissolving IL in water (AAO will float in the water after being detached), (v) removing the floated AAO with a tweezer, and (vi) immersing the transferred film/new substrate in water for >3hr to remove the IL residual.

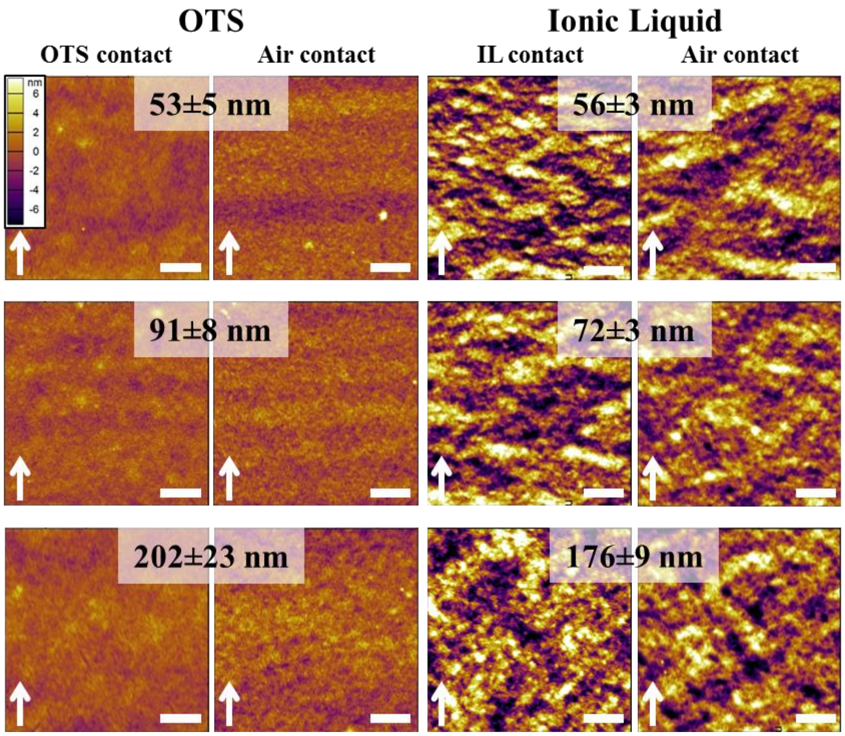

Supplementary Figure 8. AFM micrographs of top and bottom surfaces of IL-templated DPP2T-TT films vs. reference films. Tapping mode height images compare surfaces formed on substrates during coating with surfaces formed at the solution-air interface. The white arrow indicates the coating direction (all scale bars are 1 μm). We applied a triple transfer method using OTS-treated PDMS stamps to reveal the buried polymer film surface formed directly on the OTS-treated SiO_2/Si substrate. Transfer process involved delaminating OTS-coated films by placing a PDMS stamp into contact with the substrate, applying vertical pressure and rapidly peeling the film off. Then the transferred film was delaminated again by a second PDMS stamp (following the same process). Finally, the second PDMS stamp was placed into contact with another OTS-treated substrate and the film was transferred by applying vertical pressure and peeling off the stamp. On the contrary, to reveal the air contact surface of the IL-templated films for scanning via AFM, which was hidden with our primary transfer method, the thin film was floated on copious amount of water and then transferred to an OTS substrate by scooping it up. After placing the AAO/IL/film composite onto the water surface IL dissolves in water from the bottom of the composite and the film is detached facilitating the transfer process.

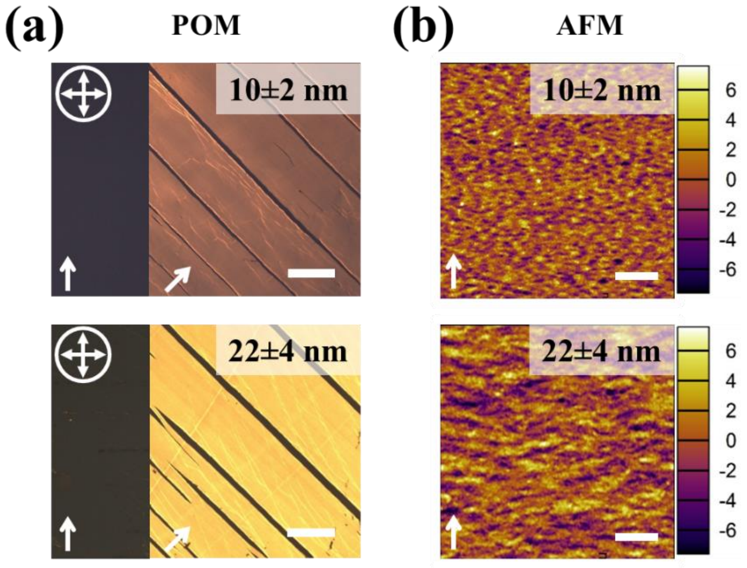

Supplementary Figure 9. DPP2T-TT thin film morphology formed on dynamic templates. (a) Cross-polarized optical microscopy images of thin DPP2-TT film coated on IL (polarizer orientation is indicated by two perpendicular arrows). The white arrow shows the coating direction (all scale bars are 100 μm). (b) Atomic force microscopy image of thin DPP2-TT film coated on IL (all scale bars are 1 μm). Cracks observed for thin films could arise from (1) high degree of polymer crystallinity, or (2) the thinness of the film that can cause lower film coverage.

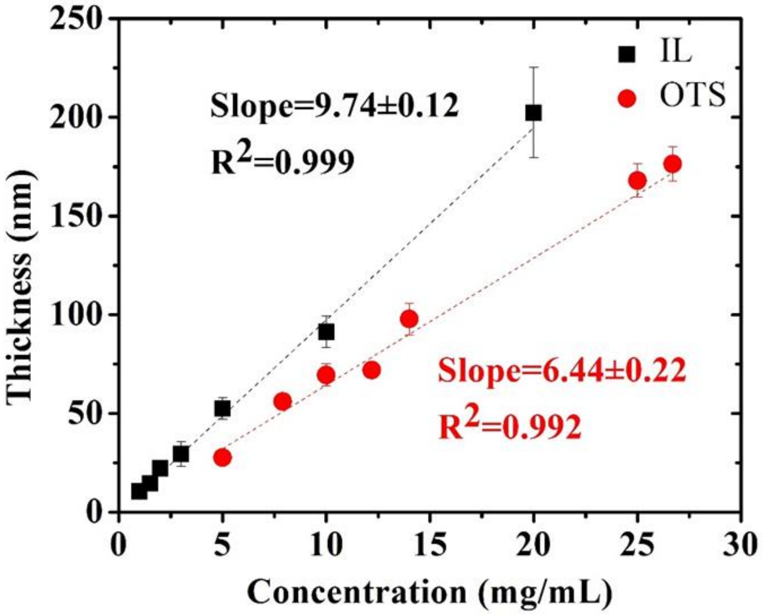

Supplementary Figure 10. Concentration vs. thickness comparing films printed on IL and OTS. Evaporation rate is calculated from this equation: $h = \frac{C}{\rho}\frac{Q_{evap}}{L.v}$ where h, C, ρ, L, v and Q_{evap} are thickness, concentration, density, meniscus length, coating speed and evaporation rate respectively[20]. Calculations (provided below) illustrates that evaporation rate on IL is approximately 50% faster than on OTS. This is consistent with experimental static advancing contact angle values of chloroform on OTS (θ_{OTS}) and on IL (θ_{IL}), where θ_{OTS} is larger than 30° but chloroform completely wets the AAO/IL hybrid (θ_{IL}~0°).

$$h = \frac{C}{\rho}\frac{Q_{evap}}{L}\nu^{-1} \rightarrow Q_{evap} = \frac{h}{C}\rho L\nu$$

$$Q_{evap,IL} = 9.74\frac{nm}{m\,g/m\,L} \times 1.49\frac{g}{cm^3} \times 1cm \times 0.5\frac{mm}{s} = 7.26 \times 10^{-5}\frac{cm^3}{s}$$

$$Q_{evap,OTS} = 6.44\frac{nm}{m\,g/m\,L} \times 1.49\frac{g}{cm^3} \times 1cm \times 0.5\frac{mm}{s} = 4.80 \times 10^{-5}\frac{cm^3}{s}$$

$$\rightarrow \frac{Q_{evap,IL}}{Q_{evap,OTS}} = 1.51$$

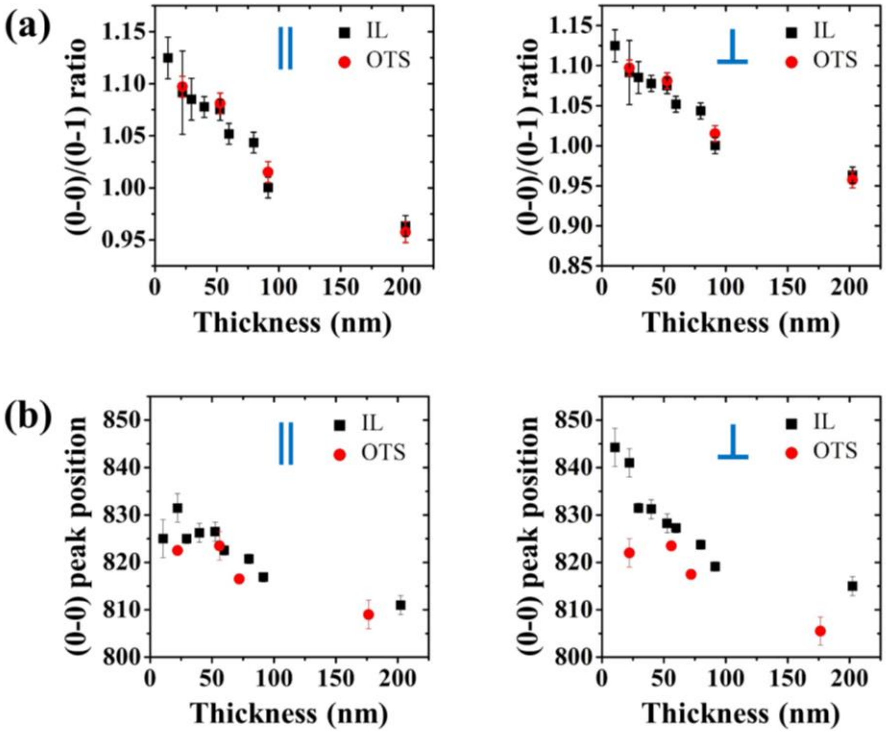

Supplementary Figure 11. Analysis of polarized **UV-Vis absorption spectra on thickness dependent vibronic peak ratio and peak position for DPP2T-TT films.** (a) 0-0 to 0-1 vibronic peak ratio as a function of film thickness with coating direction positioned parallel and perpendicular to the polarizer axis. (b) 0-0 peak position as a function of film thickness with coating direction positioned parallel and perpendicular to polarizer axis. In all plots, the black data points correspond to samples prepared on IL templates, and the red data points are from reference samples prepared on OTS substrates.

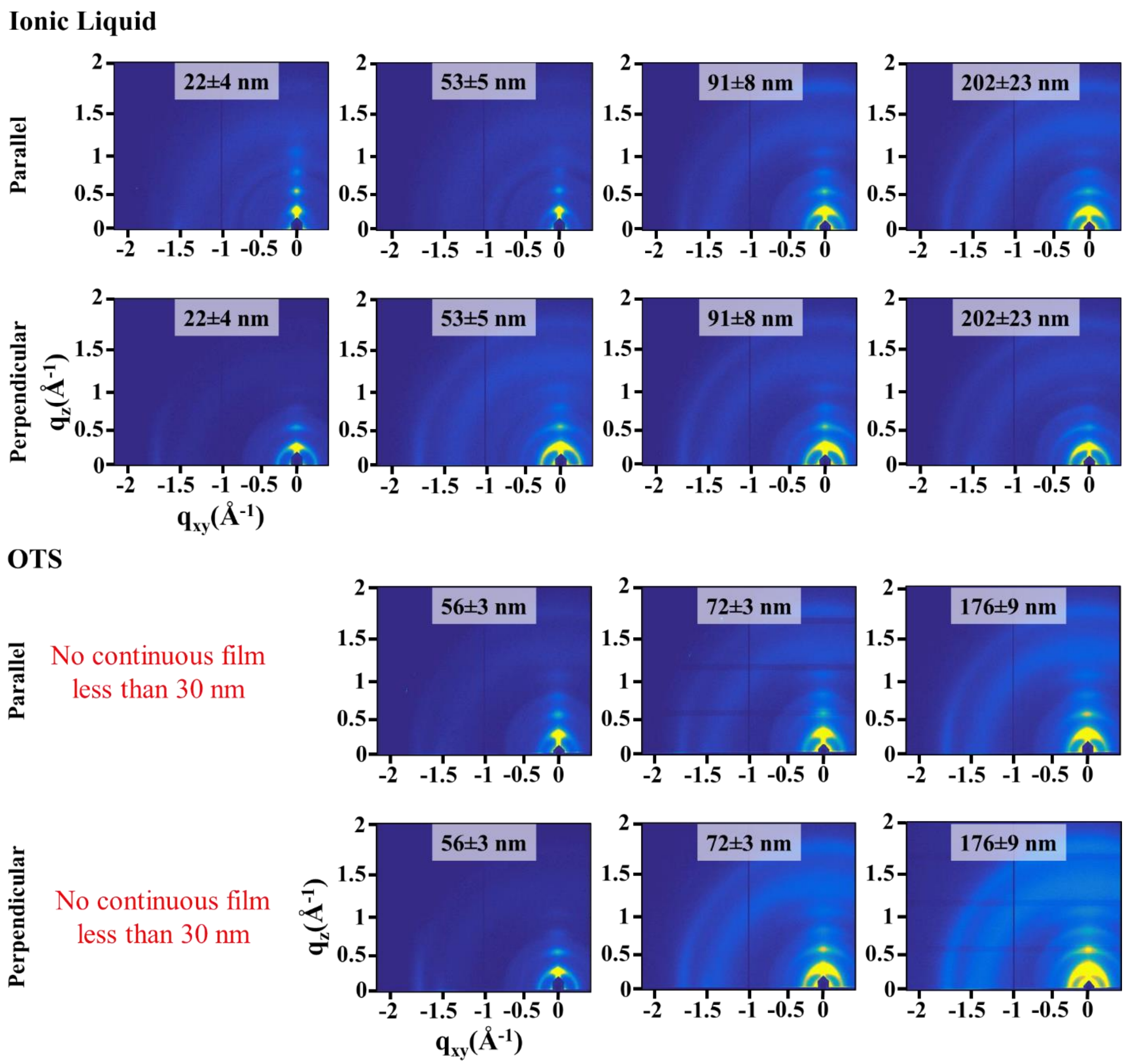

Supplementary Figure 12. GIXD images for films of various thicknesses coated on IL vs. OTS.

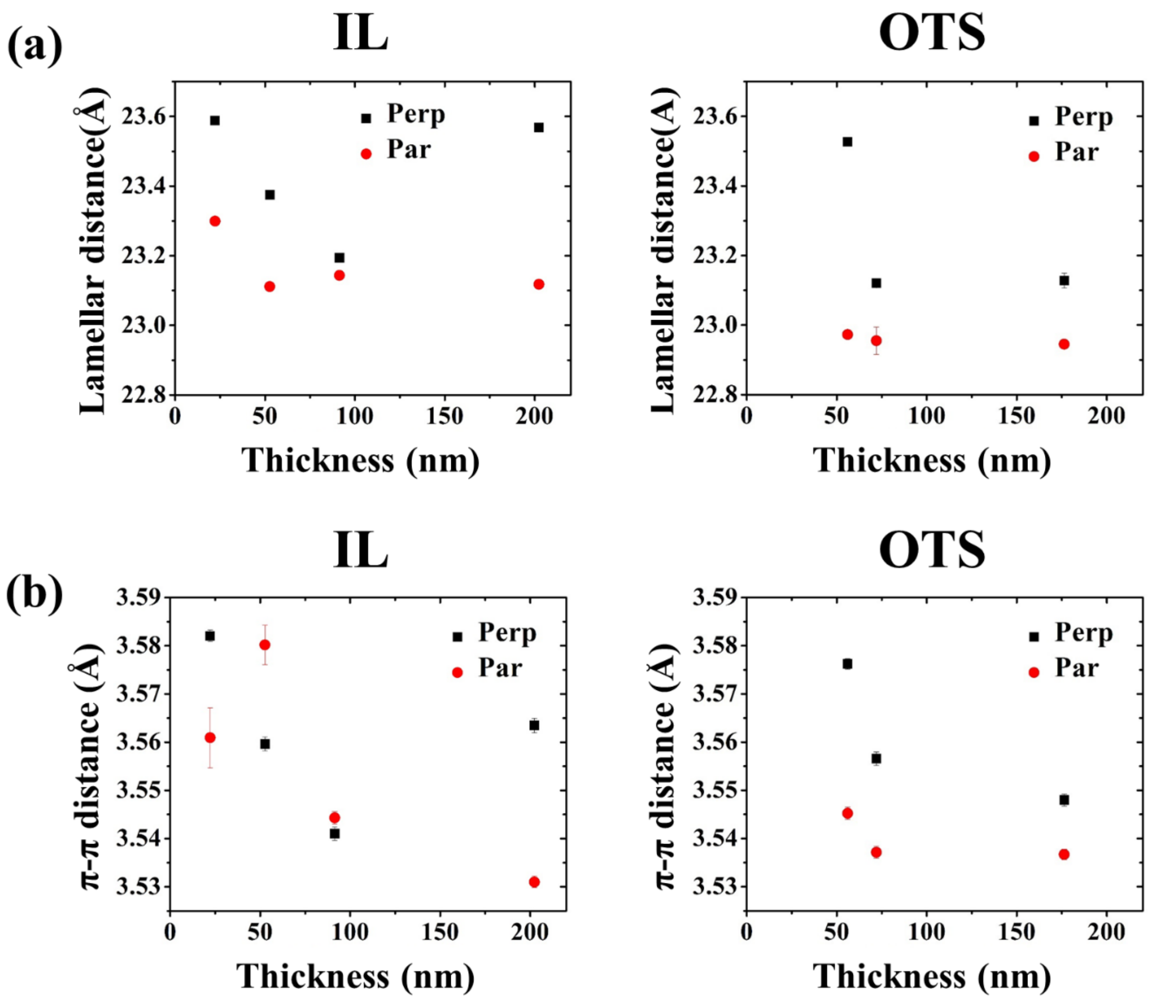

Supplementary Figure 13. GIXD peak analysis and dependence of molecular packing on film thickness. (a) Lamellar stacking distance (from (100) peak) and (b) π-π stacking distance for DPP2T-TT polymer thin films deposited on IL dynamic templates and OTS reference substrate.

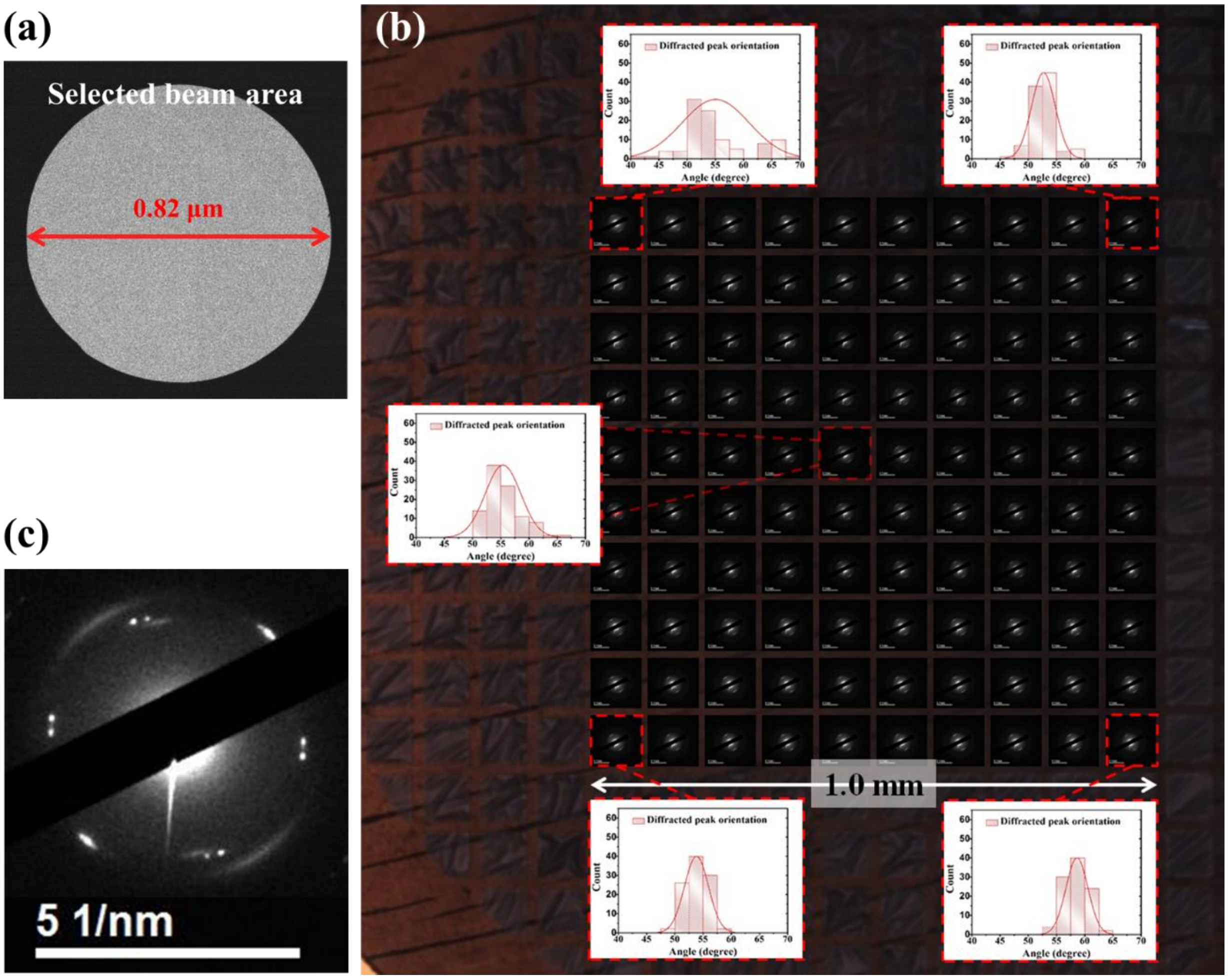

Supplementary Figure 14. TEM electron diffraction for 2D orientation mapping. (a) Selected sample area used for acquiring diffraction patterns. The thin film sample was an IL-templated DPP2T-TT thin film with an average film thickness of 10±2 nm. (b) Cross-polarized optical microscope image of the DPP2T-TT thin film on the TEM grid. Histograms of orientation distributions in five meshes across the entire film are shown. The orientation is determined in terms of the π-π peak rotation angle with respect to a fix axis arbitrarily defined. An angular spread of merely 15-20° was observed in most meshes. The deviations from mesh to mesh may be caused by solution coating or due to imperfections introduced by the film transfer process. (c) Single-crystal-like hexagonal diffraction micrographs occasionally observed during mapping.

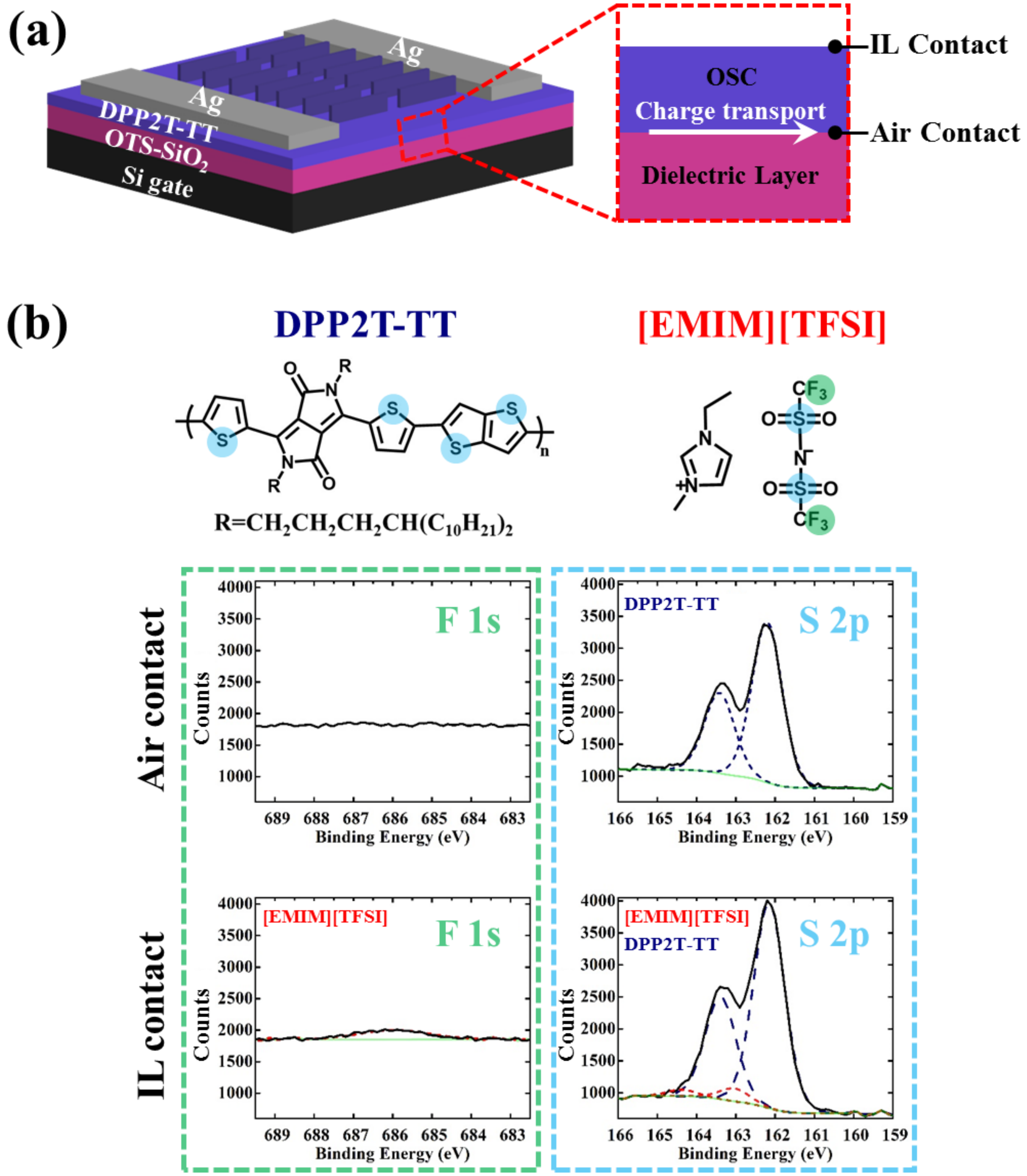

Supplementary Figure 15. IL-templated device active layer structure and composition. (a) Schematic of top-contact bottom-gate OFETs. After transferring the IL-templated thin film to the OTS-treated substrate, the polymer film's surface in contact with the IL template (IL contact) appears on the top surface; the surface near the blade and in contact with air (air contact) forms the conductive channel at the dielectric

layer interface. This configuration minimizes the effects of IL residues on the device performance. (b) High resolution X-ray photoelectron spectroscopy (XPS) spectra of the F (1s) and S (2p) regions comparing IL contact vs. air contact DPP2T-TT film with 40±5 nm thickness (coated from a 5 mg/mL solution at 0.5 mm/s). The blue (red) dashed lines in the spectra represent the deconvoluted peaks ascribed to DPP2T-TT ([EMIM][TFSI]). No fluorine signal was detected for the air contact surface, suggesting that IL is not present in the OFET conductive channel since X-ray penetration depth is <10 nm due to the employed incidence angle (90°) and charge transport occurs within the first few monolayers near the semiconductor/dielectric interface[21-22].

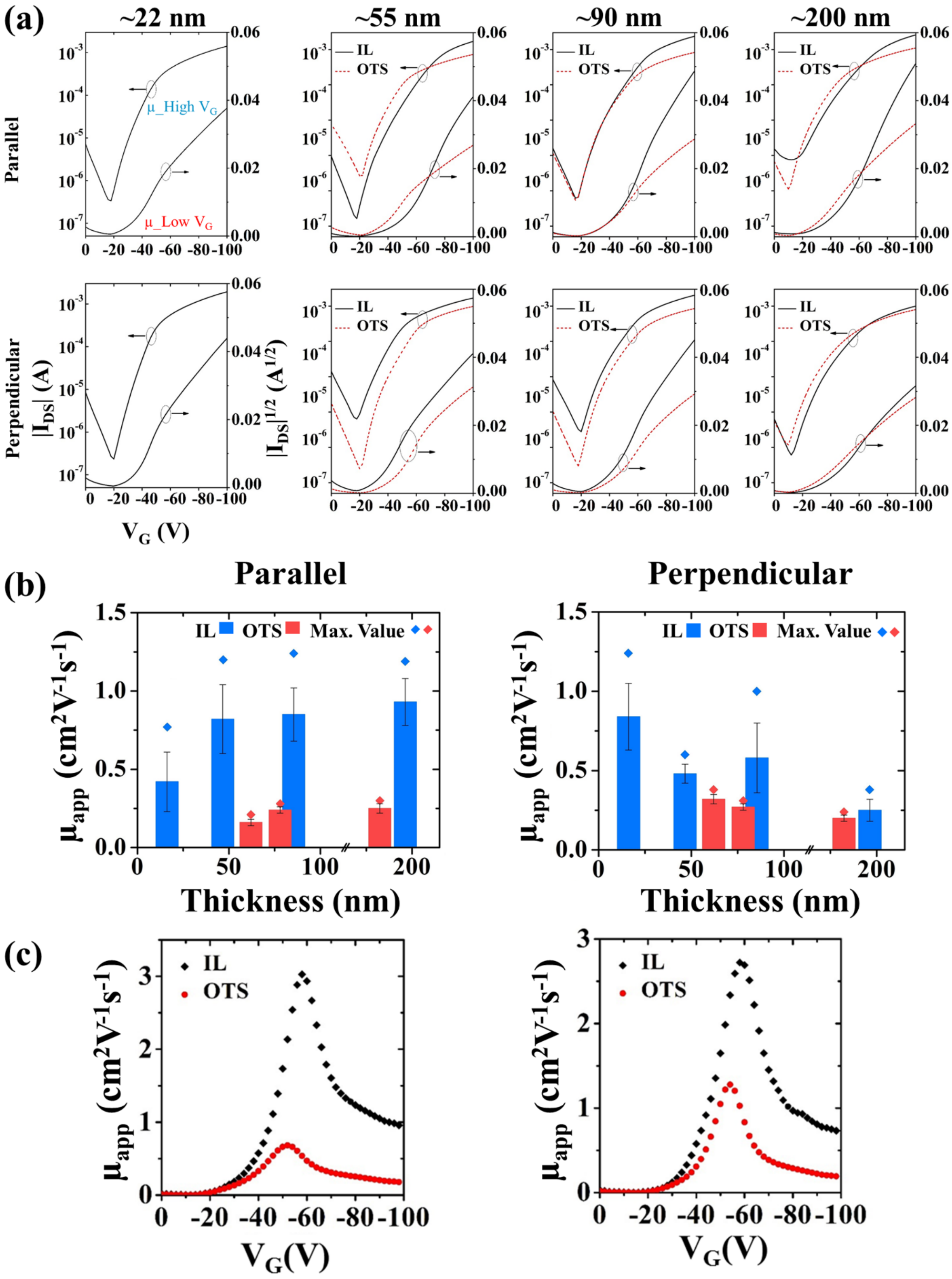

(a)
~22 nm
~55 nm
~90 nm
~200 nm
Parallel
Perpendicular
|I_DS| (A)
|I_DS|^1/2 (A^1/2)
μ_High V_G
μ_Low V_G
IL
OTS
V_G (V)
(b)
Parallel
Perpendicular
IL
OTS
Max. Value
μ_app (cm²V⁻¹s⁻¹)
Thickness (nm)
(c)
IL
OTS
μ_app (cm²V⁻¹s⁻¹)
V_G(V)

Supplementary Figure 16. OFET device characteristics. (a) Transfer curves of top contact, bottom gate OFETs fabricated fromDPP2T-TT thin films of various thicknesses, comparing IL-templated vs. reference devices (OTS) measured along both parallel and perpendicular to the coating direction. Regions of the curve used for extracting the apparent mobilities in the low and high Vg regions are shown in the first plot. (b) Comparison of average (column) and maximum (dot) mobilities between IL-templated (blue) and reference devices (red), measured parallel and perpendicular to the coating direction. The mobility values shown are apparent mobilities extracted from the high Vg region of the transfer curve in the saturation regime. The error bars on the average mobilities were standard deviations obtained from 20 independent devices. (c) Gate-voltage dependent mobility for best performing devices comparing IL-templated with reference devices (OTS), measured parallel and perpendicular to the coating direction.

Substrate	Thickness (nm)	Channel length direction w/r/t coating	Low V_G Mobility ($cm^2V^{-1}s^{-1}$) Avg (Max)	High V_G Mobility ($cm^2V^{-1}s^{-1}$) Avg (Max)	Average V_{th} (V)	Average Log (I_{on}/I_{off})	Charge Transport Anisotropy Low V_G	Charge Transport Anisotropy High V_G
IL	22±4	Parallel	0.84 (1.46)	0.42 (0.77)	-25.57	4.78	$\frac{\perp}{\parallel} = 2.13$	$\frac{\perp}{\parallel} = 1.61$
		Perpendicular	2.03 (2.72)	0.84 (1.24)	-19.36	3.61		
	53±5	Parallel	1.34 (2.26)	0.82 (1.20)	-23.59	4.13	$\frac{\perp}{\parallel} = 0.63$	$\frac{\perp}{\parallel} = 0.50$
		Perpendicular	0.97 (1.43)	0.48 (0.60)	-21.16	3.66		
	91±8	Parallel	1.57 (3.03)	0.85 (1.24)	-24.89	4.15	$\frac{\perp}{\parallel} = 0.64$	$\frac{\perp}{\parallel} = 0.81$
		Perpendicular	1.12 (1.96)	0.58 (1.00)	-23.44	4.07		
	202±23	Parallel	1.54 (2.58)	0.93 (1.19)	-25.95	4.55	$\frac{\perp}{\parallel} = 0.47$	$\frac{\perp}{\parallel} = 0.32$
		Perpendicular	0.43 (0.74)	0.25 (0.38)	-23.22	4.08		
OTS	56±3	Parallel	0.53 (0.70)	0.16 (0.21)	-28.77	4.07	$\frac{\perp}{\parallel} = 2.00$	$\frac{\perp}{\parallel} = 1.81$
		Perpendicular	1.25 (1.40)	0.32 (0.38)	-29.23	4.51		
	72±3	Parallel	0.62 (0.76)	0.24 (0.28)	-27.08	4.29	$\frac{\perp}{\parallel} = 1.34$	$\frac{\perp}{\parallel} = 1.11$
		Perpendicular	0.74 (1.02)	0.27 (0.31)	-26.81	4.30		
	176±9	Parallel	0.38 (0.63)	0.25 (0.30)	-16.47	3.77	$\frac{\perp}{\parallel} = 0.62$	$\frac{\perp}{\parallel} = 0.80$
		Perpendicular	0.31 (0.39)	0.20 (0.24)	-19.22	3.80		

Supplementary Table 2. Summary of apparent charge carrier mobilities and charge transport anisotropy for both IL-templated and reference devices (OTS) of various DPP2T-TT film thicknesses.

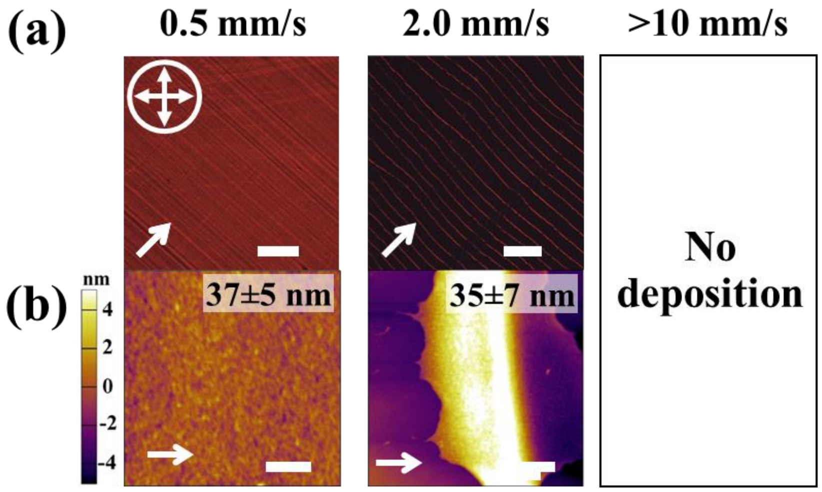

Supplementary Figure 17. Coating speed dependence of DPP2T-TT film morphology coated on OTS modified substrates. (a) C-POM and (b) tapping mode AFM micrographs coated at a wide range of speed from 0.5 mm/s to 100 mm/s. The orientation of the optical microscope cross-polarizers is shown as crossed arrows, and the white arrow indicates the coating direction. All C-POM scale bars are 100 μm and all scale bars in the AFM height images are 1 μm. Inadequate wetting prevents film deposition on OTS substrate at elevated speeds (wires are coated at 2.0 mm/s and no material is deposited at > 2.0 mm/s).

Supplementary References:

1. Wong, T.-S.; Kang, S. H.; Tang, S. K. Y.; Smythe, E. J.; Hatton, B. D.; Grinthal, A.; Aizenberg, J., Bioinspired self-repairing slippery surfaces with pressure-stable omniphobicity. *Nature* ***477***, 443-447 (2011).
2. Maestro, version 10.7, Schrödinger, LLC, New York, NY. (2016).
3. Martinez, L.; Andrade, R.; Birgin, E. G.; Martinez, J. M., PACKMOL: a package for building initial configurations for molecular dynamics simulations. *J. Comput. Chem.* ***30***, 2157-2164 (2009).
4. Keaveney, S. T.; Harper, J. B.; Croft, A. K., Computational approaches to understanding reaction outcomes of organic processes in ionic liquids. *RSC Adv.* ***5***, 35709-35729 (2015).
5. D.A. Case, R. M. B., W. Botello-Smith, D.S. Cerutti, T.E. Cheatham, III, T.A. Darden, R.E. Duke, T.J. Giese, H. Gohlke, A.W. Goetz, N. Homeyer, S. Izadi, P. Janowski, J. Kaus, A. Kovalenko, T.S. Lee, S. LeGrand, P. Li, C. Lin, T. Luchko, R. Luo, B. Madej, D. Mermelstein, K.M. Merz, G. Monard, H. Nguyen, H.T. Nguyen, I. Omelyan, A. Onufriev, D.R. Roe, A. Roitberg, C. Sagui, C.L. Simmerling, J. Swails, R.C. Walker, J. Wang, R.M. Wolf, X. Wu, L. Xiao, D.M. York and P.A. Kollman, Amber 14. (2014).
6. Sprenger, K. G.; Jaeger, V. W.; Pfaendtner, J., The General AMBER Force Field (GAFF) Can Accurately Predict Thermodynamic and Transport Properties of Many Ionic Liquids. *J. Phys. Chem. B* ***119***, 5882-5895 (2015).
7. Darden, T.; York, D.; Pedersen, L., Particle mesh Ewald: An N·log(N) method for Ewald sums in large systems. *J. Chem. Phys.* ***98***, 10089-10092 (1993).
8. Ryckaert, J.-P.; Ciccotti, G.; Berendsen, H. J. C., Numerical integration of the cartesian equations of motion of a system with constraints: molecular dynamics of n-alkanes. *J. Comput. Phys.* ***23***, 327-341 (1977).
9. Berendsen, H. J. C.; Postma, J. P. M.; van Gunsteren, W. F.; DiNola, A.; Haak, J. R., Molecular dynamics with coupling to an external bath. *J. Chem. Phys.* ***81***, 3684-3690 (1984).
10. Roe, D. R.; Cheatham, T. E., PTRAJ and CPPTRAJ: Software for Processing and Analysis of Molecular Dynamics Trajectory Data. *J. Chem. Theory Comput.* ***9***, 3084-3095 (2013).
11. McGibbon, Robert T.; Beauchamp, Kyle A.; Harrigan, Matthew P.; Klein, C.; Swails, Jason M.; Hernández, Carlos X.; Schwantes, Christian R.; Wang, L.-P.; Lane, Thomas J.; Pande, Vijay S., MDTraj: A Modern Open Library for the Analysis of Molecular Dynamics Trajectories. *Biophys. J.* ***109***, 1528-1532 (2015).
12. Meng, Y.; Zuo, J.-M., Three-dimensional nanostructure determination from a large diffraction data set recorded using scanning electron nanodiffraction. *IUCrJ* ***3***, 300-308 (2016).
13. Lewis, J. In *Fast normalized cross-correlation*, Vision interface, 1995; pp 120-123.
14. Dong, H.; Jiang, S.; Jiang, L.; Liu, Y.; Li, H.; Hu, W.; Wang, E.; Yan, S.; Wei, Z.; Xu, W.; Gong, X., Nanowire Crystals of a Rigid Rod Conjugated Polymer. *J. Am. Chem. Soc.* ***131***, 17315-17320 (2009).
15. Chen, Z.; Lee, M. J.; Shahid Ashraf, R.; Gu, Y.; Albert-Seifried, S.; Meedom Nielsen, M.; Schroeder, B.; Anthopoulos, T. D.; Heeney, M.; McCulloch, I.; Sirringhaus, H., High-Performance Ambipolar Diketopyrrolopyrrole-Thieno[3,2-b]thiophene Copolymer Field-Effect Transistors with Balanced Hole and Electron Mobilities. *Adv. Mater.* ***24***, 647-652 (2012).
16. Lei, T.; Dou, J.-H.; Pei, J., Influence of Alkyl Chain Branching Positions on the Hole Mobilities of Polymer Thin-Film Transistors. *Adv. Mater.* ***24***, 6457-6461 (2012).

17. Pople, J. A.; Untch, K. G., Induced Paramagnetic Ring Currents. *J. Am. Chem. Soc.* ***88***, 4811-4815 (1966).
18. Ma, J. C.; Dougherty, D. A., The Cation−π Interaction. *Chem. Rev.* ***97***, 1303-1324 (1997).
19. Günther, H., *NMR spectroscopy: basic principles, concepts and applications in chemistry*. Wiley: 2013.
20. Le Berre, M.; Chen, Y.; Baigl, D., From Convective Assembly to Landau-Levich Deposition of Multilayered Phospholipid Films of Controlled Thickness. *Langmuir* ***25***, 2554-2557 (2009).
21. Sirringhaus, H., Device physics of Solution-processed organic field-effect transistors. *Adv. Mater.* ***17***, 2411-2425 (2005).
22. Coropceanu, V.; Cornil, J.; da Silva, D. A.; Olivier, Y.; Silbey, R.; Bredas, J. L., Charge transport in organic semiconductors. *Chem. Rev.* ***107***, 926-952 (2007).
